# The Potential of Modified and Multimeric Antimicrobial Peptide Materials as Superbug Killers

**DOI:** 10.3389/fchem.2021.795433

**Published:** 2022-01-10

**Authors:** Tamara Matthyssen, Wenyi Li, James A. Holden, Jason C. Lenzo, Sara Hadjigol, Neil M. O’Brien-Simpson

**Affiliations:** ^1^ ACTV Research Group, The University of Melbourne, Melbourne Dental School, Centre for Oral Health Research, Royal Dental Hospital, Melbourne, VIC, Australia; ^2^ Centre for Oral Health Research, The University of Melbourne, Melbourne Dental School, Royal Dental Hospital, Melbourne, VIC, Australia

**Keywords:** antimicrobials1, peptides2, multimerisation3, superbugs4, AMPs5

## Abstract

Antimicrobial peptides (AMPs) are found in nearly all living organisms, show broad spectrum antibacterial activity, and can modulate the immune system. Furthermore, they have a very low level of resistance induction in bacteria, which makes them an ideal target for drug development and for targeting multi-drug resistant bacteria ‘Superbugs’. Despite this promise, AMP therapeutic use is hampered as typically they are toxic to mammalian cells, less active under physiological conditions and are susceptible to proteolytic degradation. Research has focused on addressing these limitations by modifying natural AMP sequences by including e.g., d-amino acids and N-terminal and amino acid side chain modifications to alter structure, hydrophobicity, amphipathicity, and charge of the AMP to improve antimicrobial activity and specificity and at the same time reduce mammalian cell toxicity. Recently, multimerisation (dimers, oligomer conjugates, dendrimers, polymers and self-assembly) of natural and modified AMPs has further been used to address these limitations and has created compounds that have improved activity and biocompatibility compared to their linear counterparts. This review investigates how modifying and multimerising AMPs impacts their activity against bacteria in planktonic and biofilm states of growth.

## Introduction

As drug resistant bacterial infections increase globally, the age of antibiotics is reported to be coming to an end (O’Neill, 2016). Antibiotics are crucial to modern healthcare as they are used not only for treating bacterial infections, but for making important medical procedures and treatments requiring immune suppression possible (Interagency Coordination Group on Antimicrobial Resistance, 2019). The World Health Organisation (WHO) estimates that, globally, over 700,000 deaths per year are due to drug resistant diseases, with this number predicted to rise to 10 million deaths by 2050 if nothing is done to change the current situation (O’Neill, 2016). The need for alternative antibacterial therapeutics that are less likely to induce antimicrobial resistance are therefore needed more than ever.

**FIGURE 1:**
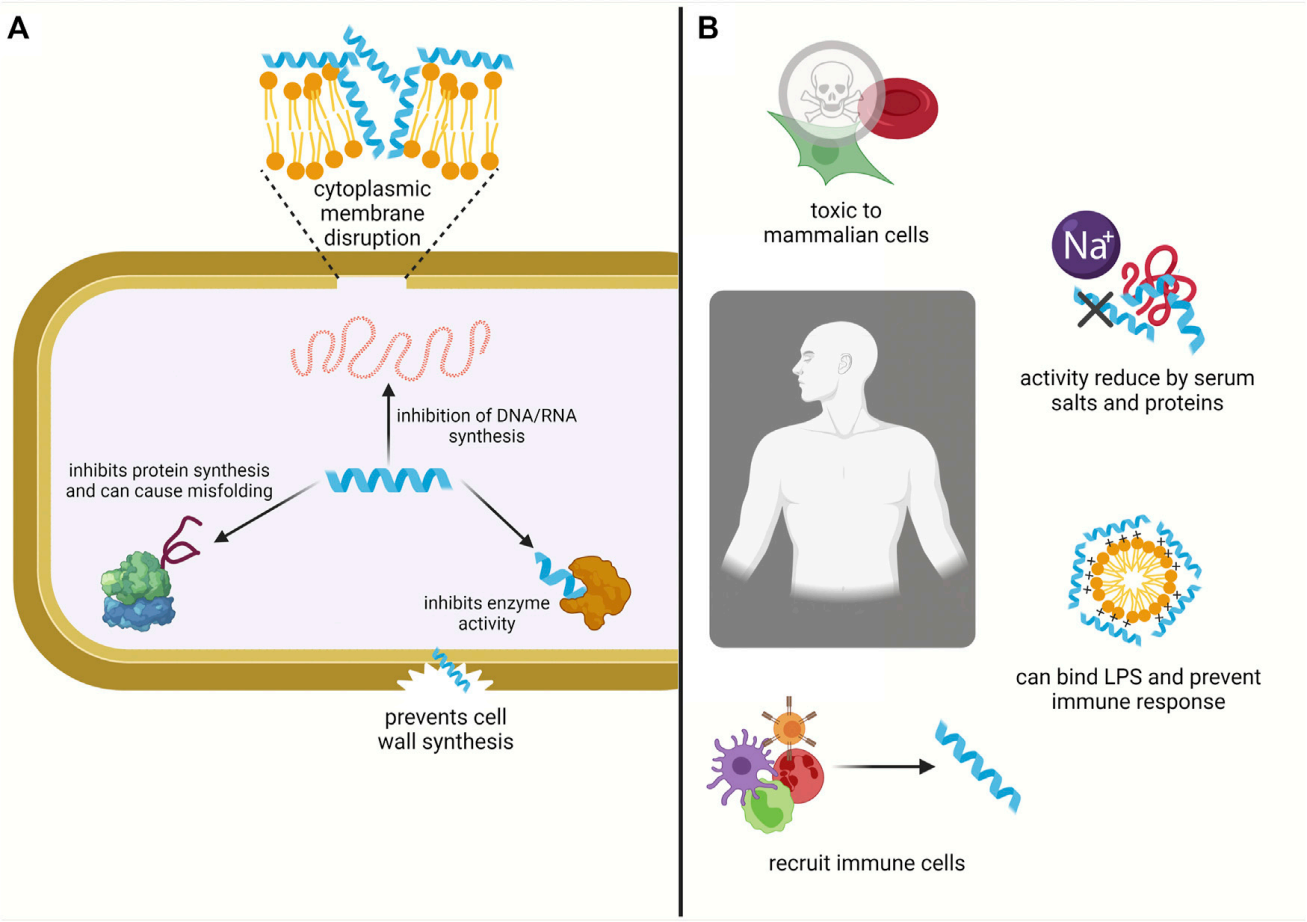
AMPs interact with both bacterial and mammalian cells. AMPs form amphipathic secondary structures upon interaction with bacterial membrane. They kill bacteria by either disrupting the inner cytoplasmic membrane or acting on internal targets **(A)**. Within the body they can also prevent inflammation, e.g., by binding and neutralising the lipopolysaccharides from Gram-negative bacteria and they can modulate the innate immune response by recruiting immune cells. However, their activity is reduced under physiological conditions due to the presence of serum salts and proteins and they are susceptible to proteolysis. AMPs can also be cytotoxic towards mammalian cells and exhibit haemolytic activity **(B)** (Created with BioRender.com).

A promising avenue of research stems from naturally occurring antimicrobial peptides (AMPs) that have a broad spectrum of activity (Zasloff, 2002). AMPs are found in all living organisms and are also referred to as host defense peptides, or defensins (Campopiano et al., 2004; Qian et al., 2018; Zasloff, 2002). AMPs have been shown to directly kill bacteria by disrupting the cellular membrane or acting on intracellular targets. They can also modulate the body’s inflammatory and innate immune response, recruiting immune cells to promote wound healing or angiogenesis (Figure 1) (Dong et al., 2018; Hancock and Sahl, 2006; Afacan et al., 2012; Zasloff, 2002).

AMPs have demonstrated strong antimicrobial activity both *in vitro* and *in vivo* and are of special interest as they do not readily induce antimicrobial resistance (Zasloff, 2002). According to the Antimicrobial Peptide database (APD3), at least 3,000 active natural AMPs have been discovered in various organisms (Wang G. et al., 2016). Despite the large number of known natural AMPs, their clinical application has been constrained by their various limitations. These limitations include cytotoxicity, susceptibility to protease degradation and a short *in vivo* half-life (Hancock and Sahl, 2006). Various studies have revealed that certain amino acids are more prevalent than others among AMPs and that charge, hydrophobicity, and overall structure affect the level of antimicrobial activity (Kumar et al., 2018). Manipulating these inherent properties of AMPs has helped overcome some of their limitations. It has also been demonstrated that multimerisation of AMPs is another technique that can improve their activity and biocompatibility to help overcome issues of cytotoxicity and stability (Giuliani and Rinaldi, 2011; Sobczak et al., 2013; Ortega et al., 2020). This review provides a summary of some of the modifications made to AMPs that increase their activity and aims to highlight their effects on AMP structure. The review also explores different multimerisation strategies and the features of multimerised compounds that affect activity and biocompatibility.

## Properties and Limitations of Antimicrobial Peptides

### General Properties

AMPs show antimicrobial activity towards both Gram-negative and Gram-positive bacteria, parasites, fungi and some viruses (Gordon et al., 2005). Natural AMPs can vary greatly in length, from 2 to >200 amino acids in length. However, the majority are 10–50 amino acid residues in length, at least 30% of their residues are hydrophobic, have an overall positive charge and form amphipathic secondary structures of α-helices, β-sheets, looped peptides or extended structures upon interaction with a lipid membrane (Hancock and Sahl, 2006; Nguyen et al., 2011; Takahashi et al., 2013; Wang J. et al., 2016). Modifying AMP size, sequence, net charge, conformation, structure, hydrophobicity and amphipathicity can all affect AMP antimicrobial activity and specificity for certain cell types (Hong, 2001; Dennison et al., 2005; Gagnon et al., 2017; Chen W. et al., 2019).

### Mode of Action

**FIGURE 2:**
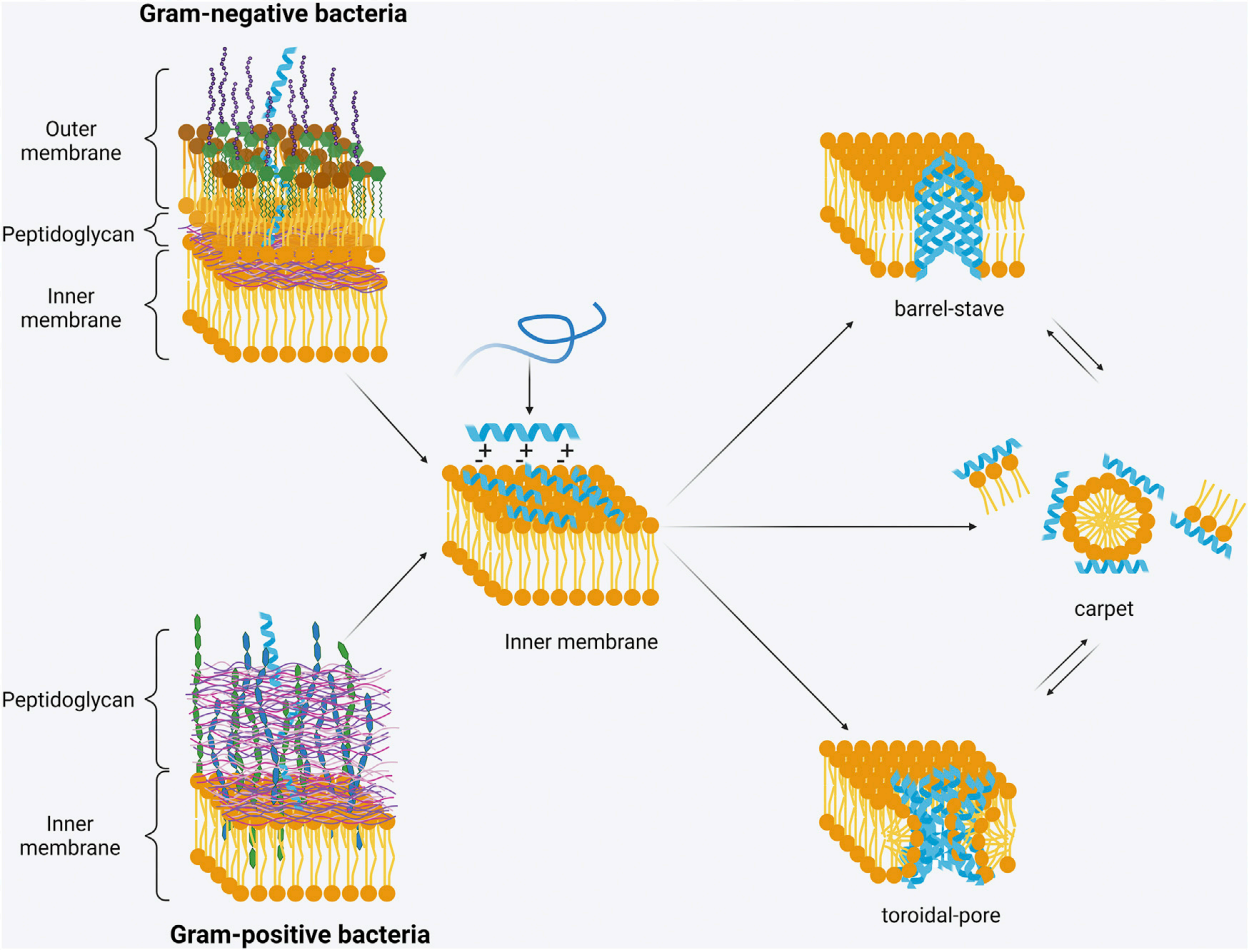
Permeabilisation of the inner membrane. Upon interaction with the negatively charged bacterial membrane AMPs form a secondary structure. Once a threshold concentration has been reached, they insert into the membrane with three models being the most widely accepted – barrel-stave, toroidal pore or carpet model (Created with BioRender.com).

The overall positive net charge of AMPs attracts them to the negatively charged surface of bacteria where they interact and transit through either the outer-membrane or peptidoglycan layers of Gram-negative or Gram-positive bacteria, respectively, and then embed in to the inner/cytoplasmic membrane. Once a certain threshold concentration of AMPs is reached at the inner membrane, AMPs insert themselves into the membrane through hydrophobic and electrostatic interactions (Hale and Hancock, 2007). These membrane-active AMPs are believed to form pores in the inner membrane, with two popular modes of action having been proposed – toroidal-pore and barrel-stave (Figure 2) (Avci et al., 2018). In the barrel-stave model, peptides interact to form a structure within the membrane to create a channel similar to that of a membrane protein ion channel. In the toroidal pore model, however, there is little to no peptide-peptide interaction. Rather, the peptides affect the curve of the lipid bilayer by interacting with the lipid hydrocarbons and headgroups to disrupt the separation of the polar and non-polar parts (Wimley, 2010).

It is also proposed that some AMPs permeabilise the membrane through non-pore forming models, such as the carpet model (Figure 2). This model suggests that the peptides cover the bacterial membrane surface and then reorient themselves, so that the hydrophobic face is towards the lipids, and the hydrophilic part faces the phospholipid head group. The peptides then cause the membrane to disintegrate by disrupting the bilayer curvature (Shai, 2002). Membrane disruption is the predominant mode of action for many AMPs. However, it is not always essential for activity. Some AMPs translocate across the membrane and act on internal targets. For example, human β-defensin 3 (hBD3) binds lipid II and inhibits cell wall synthesis, resulting in bacterial death (Sass et al., 2010). On the other hand, proline-rich AMPs, such as pyrrhocoricin, can inhibit DnaK or bind to the 70S ribosome. This respectively leads to protein misfolding or inhibition of protein translation and, in both cases, to cell death (Kragol et al., 2001; Krizsan et al., 2014). The definition and mode of action of proline-rich AMPs has been recently reviewed by Welch et al. (2020). AMPs have also been shown to cause aggregation of anionic intracellular molecules such as DNA and ribosomes (Chongsiriwatana et al., 2017; Zhu Y. et al., 2019). It is proposed that flocculation of these molecules can lead to inhibition of growth or cell death and is an important mechanism representative of the breadth of AMP activity. Other internal/cytosolic mechanisms of action are inhibition of DNA/RNA synthesis (buforin II), protein synthesis (pleurocidin), cell division (indolicidin), and enzymes (histatin 5) (Park et al., 1998; Subbalakshmi, 1998; Gusman et al., 2001; Patrzykat et al., 2002).

### Effect of Structure, Hydrophobicity, Amphipathicity and Charge on Antimicrobial Peptide Activity and Selectivity

#### Antimicrobial Peptide Structure

Modification of certain predominant features of AMPs (amino acid length, hydrophobicity, charge and amphipathicity) can lead to changes in their activity. An *in-silico* study of 100,000 9-mer peptides also indicated that AMP structure impacts its antimicrobial activity (Cherkasov et al., 2009). From this virtual library, 200 peptides were tested *in vitro* against *Pseudomonas aeruginosa*. Interestingly, this showed that peptides with very similar hydrophobicity, amphipathicity and charge, had varying antibacterial activity towards *P. aeruginosa*. The two lead compounds KRWWKWIRW and KRWWKWWRR had high antibacterial activity whereas AIRRWRIRK and WVRFVYRYW were virtually inactive while being considered to have similar amino acid composition to the lead compounds by the authors. It was suggested that the order of amino acids within the sequence affects antimicrobial activity and that this change in activity is likely due to the different final structures formed by these peptides. Unfortunately, the study did not determine the structure of these compounds, so it is unclear whether the increased activity was due to the final structures formed or due to other aspects such as overall charge and hydrophobicity. As many naturally occurring AMPs form α-helices or β-sheets and these structures have different impacts on AMP activity, the conclusion that the order of the amino acid sequence would affect this is not surprising. Non-covalent interactions between residues within a sequence determine what secondary structures can be formed. As such, disrupting or changing a specific sequence can alter their possible interactions, and hence formation of an α-helix or β-sheet.

The largest and most well studied group of AMPs are those that form α-helices and many β-sheet AMPs have been discovered in animals and plants (Hancock and Lehrer, 1998; Koehbach and Craik, 2019). Both α-helical and β-sheet AMPs are believed to exert their activity via membrane disruption but have been shown to have differing specificities. In general, the propensity to form an α-helix results in more potent activity whilst many β-sheet peptides are stabilised by disulfide bonds and their activity correlated to their amphipathicity. For example, two peptides derived from PGLa (all peptide sequences found in Supplementary Appendix A) shared similar hydrophobicity and the same charge but differed in their secondary structure (Blazyk et al., 2001; Jin et al., 2005). One adopted an α-helix ((KIAGKIA)_3_-NH_2_) whilst the other formed a β-sheet ((KIGAKI)_3_-NH_2_). Both these peptides had similar antibacterial activity, however the β-sheet was not haemolytic and the α-helical structure better at inducing leakage in *E*. *coli* cells. This similarity in antibacterial activity but difference in haemolytic activity and membrane leakage indicate differences in the mode of action of these two structures.

The comparison of a number of β-sheet peptides, revealed a correlation between their amphipathicity and activity as well as a conserved C-terminal cysteine (Edwards et al., 2016) The most amphipathic compounds were tachyplesin-1 and protegrin-1, closely followed by arenicin-3 and polyphemusin-1 with gomesin and thanatin the least amphipathic. Overall, the more amphipathic compounds exhibited better activity towards both Gram-negative and Gram-positive bacteria (e.g., MIC range of 0.016–0.25 μg/ml for tachyplesin-1 compared to 2–256 μg/ml for thanatin). The role of the conserved C-terminal cysteine found in these AMPs was not explored in this study but another study that deleted the cysteines that formed the first disulfide bond of an arenicin-3 analogue and named N6 found that it did not significantly impact its antimicrobial activity (Yang et al., 2017). β-sheets are often stabilised by disulfide bonds which offers such AMPs better proteolytic stability (Koehbach and Craik, 2019). The role of the conserved cysteine found in the aforementioned AMPs may hence be limited to overall peptide stability. To further understanding of AMP activity, a comparative study of the proteolytic stability of α- and β-structures that have similar hydrophobicity, charge, and amino acid sequence to determine whether β-sheet-containing peptides have more to offer than reduced haemolytic activity, is needed. Additionally, comparison of whether the number of β-turns within a compound affect its activity could be of interest to rational AMP design and optimisation.

The role of helicity and its correlation with AMP antibacterial activity has been explored by replacing one or more of the residues of an helical peptide derived from CP2600 (peptide *p*) with their d-amino acid counterparts (Huang et al., 2014). This resulted in analogues with decreased helicity but varying antimicrobial activity. Substituting 1, 2 or 3 residues with their d-amino equivalents not only increased antimicrobial activity towards the Gram-negative *E. coli* and *P. aeruginosa* bacteria compared to the parent peptide, but also reduced their haemolytic activity. However, substituting 4 or more residues resulted in a decrease in antimicrobial activity. When tested against the Gram-positive bacteria *S. aureus*, it was only substitution of K14 that showed improved antimicrobial activity, all other analogues showed similar or decreased activity. However, against Gram-positive *Bacillus subtilis*, substituting 1, 2, 3 or 4 residues with d-amino acids resulted in analogues with improved antimicrobial activity. Scanning electron microscopy (SEM) images of *P. aeruginosa* and *S. aureus* treated with the lead analogue (L12_D_/L20_D_) indicated that the peptide interacted with and damaged the bacterial cell membrane. As L12_D_/L20_D_ appears to exert its activity on the cell membrane, the difference in activity between the two Gram-positive strains may be due to differences in their membrane composition. According to a review on cell membrane composition by Sohlenkamp and Geiger, the major membrane lipids in *S. aureus* are phosphatidylglycerol (PG), cardiolipin (CL), lysyl-phosphatidylglycerol (LPG) and glycophospholipid (GPL) (Sohlenkamp and Geiger, 2015). In addition to these membrane lipids, *B. subtilis* also contains phosphatidylethanolamine (PE) and glycolipid (GL). According to this review *E. coli* and *P. aeruginosa* membranes also contain *p*E. AMPs may interact with specific membrane lipids in specific ways which may account for their varying specificity for different bacteria. An exploration of bacterial membrane composition and susceptibility to AMPs could help in AMP design and specific targeting but is outside the scope of this review. In general, amongst the analogues, decreasing helicity resulted in weaker antimicrobial activity. However, analogues with 1, 2 or 3 d-amino substitutions and reduced helicity exhibited improved activity compared to the parent peptide which was the most helical. In addition to improved activity, the aforementioned d-amino substituted analogues also exhibited lower haemolytic activity than the parent peptide which resulted in all of the analogues having higher therapeutic indices. Substitution with d-amino acid residues also affected the overall hydrophobicity of these peptides. A decrease in hydrophobicity was seen to be correlated with an increase in the number of d-amino substitution in this series of analogues which may account for the observed decrease in haemolytic activity. Peptides with multiple d-amino substitutions and hence lower helicity were the least haemolytic, indicating that the degree of helicity also contributed to toxicity.

More recently, the role of helicity was further explored using peptoid 1, a peptoid (residues with the side-chain moiety connected to the backbone nitrogen rather than the α-carbon of the amino acid) mimic of magainin 2 (Patch and Barron, 2003). This peptoid was made using N-(4-aminobutyl)-glycine (*N*Lys), a peptoid analogue of lysine (Lys (K)), and (S)-N-(1-phenylethyl)amine (*N*spe), an analogue of phenylalanine (Phe (F)) that acts as a helix inducer (Nam et al., 2020). Peptoid 1 was reported to be fully helical and a non-helical peptoid 2 was also created by substituting all *N*spe with N-(phenylmethyl)glycine (*N*pm). A series of 17 peptoids were also made by substituting 1 or more *N*spe at different positions with *N*pm and these peptoids exhibited varying degrees of helicity but similar hydrophobicity to peptoids 1 and 2. The fully helical peptoid 1 was the most potent but also displayed strong haemolytic activity whilst peptoid 2 was the least active and the least haemolytic. The antimicrobial activity and toxicity of all other moderately helical peptoids fell within a range between peptoid 1 and 2 but there was no explicit correlation between helicity and activity determined by the authors. However, of interest was the degree to which helices were formed in different environments. The degree of helical fold of peptoids 1, 16 and 17 were compared using circular dichroism (CD) spectroscopy in three different environments: aqueous buffer, lipid vesicles mimicking *E. coli* membranes and vesicles mimicking erythrocyte membranes. Both peptoid 16 and 17 had 4 *N*spe substituted with *N*pm but at different positions. Peptoid 16 had substitutions at the C- and N-terminus whereas peptoid 17 had substitution in the middle which was shown to affect the flexibility of these peptoids. Peptoids 1 and 16 displayed strong and weak CD intensity, respectively, which did not vary between different environments. Peptoid 17, however, which showed a weaker degree of helicity than peptoid 16 in aqueous buffer exhibited a much greater degree of helicity in the bacterial membrane mimic environment. A comparison of their MICs showed that against *E. coli*, peptoid 1 was the most potent (3.1 µM) followed by peptoid 17 (6.3 µM) with peptoid 16 the weakest (12.5 µM). Peptoids 16 and 17 were also much less haemolytic than peptoid 1 with peptoid 17 having the greatest selectivity index (calculated as HC_10_/MIC in *E. coli*). The flexibility of peptoid 17 was considered to be the reason for its selectivity towards the bacterial membrane. Such conformational flexibility can also be found in nature and the ability of this feature to improve AMP activity and selectivity has been more thoroughly explored in several papers (Juretić and Simunić, 2019; Liu et al., 2013; Rončević et al., 2018). Whilst the degree of helicity is important for AMP activity, the environment in which the AMP forms this structure, and its flexibility should be considered as equally important for AMP design.

#### Hydrophobicity

An earlier study on a variant of CP2600 explored how single amino acid substitution of this peptide could alter hydrophobicity and subsequently, activity. The variant was created via DNA mutagenesis and named V_681_ (Zhang et al., 1998). Valine (Val) 13 of this peptide was then substituted with lysine (V13Kʟ) and was shown to have good antimicrobial activity and a higher therapeutic index (TI) (Chen et al., 2005). Val 16 of V13Kʟ was then substituted with various amino acids and it was found that a single amino acid substitution could greatly alter the peptide’s overall hydrophobicity. The analogue V16L (Val 16 of V13K_L_ substituted with Leucine (Leu)), was the most hydrophobic as shown by its increased retention time when analysed using RP-HPLC. This resulted in V16L being slightly more active than V13Kʟ but it was also considerably more haemolytic, causing 53.9% haemolysis compared to 28.3%, respectively (Tan et al., 2014). Substitution with less hydrophobic amino acids and at hydrophobic positions other than Val 16 may result in analogues with reduced haemolysis. In this same study, V16A (substitution with Alanine (Ala)) was less hydrophobic than V13Kʟ, had the same MIC towards a clinical isolate of *E. coli* and *P. aeruginosa* ATC2853 but was less haemolytic. It caused only 14.3% haemolysis. This shows that the issues of cytotoxicity may be addressed by using different hydrophobic amino acids to maintain amphipathicity of an AMP but reduce the overall hydrophobicity.

This concept of substituting hydrophobic residues with other hydrophobic residues to observe their effects on activity and toxicity has been explored using the platelet factor IV derived AMP C18G (Saint Jean et al., 2018). A series of peptides were made by replacing Leu of C18G with Phe, isoleucine (Ile), Val or α-aminoisobutyric acid (Aib). The change of Leu to Phe or Ile did not change the MIC of the peptide when tested against *E. coli*, *S. aureus*, *Salmonella enterica*, *P. aeruginosa* or *Staphylococcus epidermidis*. However, the change to Val increased the MIC values against all bacteria tested and the change to Aib increased the MIC even more. This agrees with the previously discussed study in which substitution of Val with Leu resulted in a decreased MIC. A similar trend was observed in lipid vesicle and bacterial membrane permeabilisation studies in which C18G displayed the highest activity (∼95% lipid vesicle content leakage at 1.5 µM), followed by the Leu → Phe (∼35% at 1.5 µM) and Leu → Ile (∼40% at 1.5 µM) variants that also showed considerable permeabilisation. The Leu → Val and Leu → Aib showed some, albeit considerably less, permeabilisation (lipid vesicle content leakage ∼30% for Val and Aib at 1.5 µM and ∼35% at 15 µM compared to 100% for Leu, Phe and Ile also at 15 µM). C18G and Leu → Phe did the most damage to the *E. coli* outer membrane followed by the Leu → Ile variant. The Val and Aib were also capable of causing some damage but to a much lesser extent. However, C18G was the only peptide that showed any considerable damage to the inner membrane of *E. coli*. C18G was also the most toxic to HEK-293 cells (10% cell viability at 15 µM) compared to ∼50% viability for Phe substitution, ∼65% for Aib, ∼80% for Ile and ∼88% for Val all at 15 µM. Although, at 1.5 µM (close to the MIC for C18G), cell viability ranged from ∼80–100% for all peptides. It was postulated that the variation in antimicrobial activity and cell toxicity observed was not solely due to overall hydrophobicity but was affected by the side chain length and bulkiness of the hydrophobic residues. Order of side chain length was considered to be Phe > Ile > Leu > Val > Aib and for bulkiness Phe > Leu > Ile > Val/Aib. Substitution of Leu with Phe or Ile did not significantly impact activity, indicating that the increased side chain bulk of Phe had little effect on activity. However, substitution with Val showed that a significant decrease in side-chain length and bulk decreased activity considerably which was even more pronounced for analogues with the shorter chain Aib substitutions. Amino acids with bulky side-chains, such as tryptophan (Trp), can disrupt the equilibrium of bacterial membranes and have been shown to cause disorganisation of the lipopolysaccharide (LPS) leaflet found in Gram-negative bacteria. Isothermal calorimetric titration of LPS with Trp containing peptides demonstrated that an exothermic process occurred first, followed by an endothermic reaction (Shang et al., 2016). This resulted in disruption of the outer membrane of Gram-negative bacteria as the peptide buried into the LPS leaflet. Shifts in the emission spectra of Trp fluorescence in the presence of a negatively charged membrane has also been observed and shows that Trp residues bury deeply into the membrane (Bi et al., 2013). Once peptides bury into the lipid bilayer, the bulky side chain of Trp can then disrupt the hydrophobic interactions of the acyl chains and hence the membrane structure (Chan et al., 2006). This ability to traverse the outer membrane, bury into the lipid bilayer and disrupt the entropy of the membrane, can increase the overall antimicrobial activity of peptides containing residues with bulky side-chains.

Increasing overall hydrophobicity has also been shown to increase activity and haemolysis, although the evidence is less distinct (Tan et al., 2014). As reviewed by Moret and Zebende, different methods have been developed for determining the level of hydrophobicity of amino acid residues with each method ordering them differently (Moret and Zebende, 2007). However generally, alanine is the least hydrophobic with valine almost always more hydrophobic than methionine. The order of hydrophobicity of leucine, isoleucine, phenylalanine, tryptophan, tyrosine, and cysteine then vary depending on the method used. It is important to note that pH also affects hydrophobicity. The amino acid residues that are chosen to increase the hydrophobicity of an AMP, and their position within the final construct, can determine the extent to which activity and toxicity is affected. If a certain residue increases hydrophobicity but also disrupts other key features such as amphipathicity, helicity or net charge, then increased activity may not be observed. Additionally, replacing a less hydrophobic residue with a more hydrophobic one will not necessarily result in increased activity. Some studies suggest this may be due to how residue side chains insert and interact with the lipid bilayer of bacterial membranes (Saint Jean et al., 2018). In addition to changing hydrophobicity and helicity, changing the amphipathic profile of an AMP also affects its activity.

#### Amphipathicity

To explore how amphipathicity affects activity and toxicity, alanine 8 on the polar face of AR-23 (an AMP from *Rana tagoi*, Brown frog) was substituted with positively charged Arg (R) (named A (A8R)), which increased its amphipathicity (Zhang et al., 2016). Conversely, Ala1 and Ile 17 on the non-polar face was substituted with Arg (A (A1R)) and Lys (K) (A (I17K)) respectively. A (A1R) had the same degree of amphipathicity as the parent peptide, but A (I17K) was slightly less amphipathic than the parent AR-23 peptide. The more amphipathic A (A8R) analogue showed increased antimicrobial activity towards *S. aureus* and *E. coli* as well as decreased haemolytic activity. Conversely, A (I17K) showed no change or a decrease in activity towards *E. coli* and *S. aureus* respectively but was much less haemolytic than both AR-23 and A (A8R). As for A (A1R), which displayed the same degree of amphipathicity as AR-23, it had the same MIC as A (A8R) towards *E. coli* but towards *S. aureus* its MIC was the same as AR-23. It was also less haemolytic than AR-23 but slightly more so than A (A8R). Considering the changed amphipathicity of these analogues and the effect on the MIC values, an increase in amphipathicity appeared to increase the antimicrobial activity of the peptide.

Other analogues that consisted of a mix of more than 1 of the above substitutions were also made. These analogues varied in their degree of amphipathicity and activity towards *S. aureus* and *E. coli*, but all of them were less haemolytic than AR-23. Along with the single substitution analogues, they also caused more membrane damage to *E. coli* cells than AR-23 (except for A (A8R, I17K)). However, they all caused less membrane damage to *S. aureus* compared to AR-23. These substitutions also affected the overall hydrophobicity, helicity, and charge on these AMPs, making it difficult to determine the exact effect changes to amphipathicity have on antibacterial activity and toxicity. Although A (A8R) and A (A1R, A8R) both had an increased amphipathic profile and lower haemolytic activity, they differed in their antimicrobial activity and α-helical content. The A (A8R) analogue had a higher α-helical content than both the parent peptide and A (A1R, A8R) in 50% v/v TFE. It also had lower MIC values than AR-23 towards *E. coli* and *S. aureus*, was less active than A (A1R, A8R) towards *E. coli* but more active towards *S. aureus*. The degree of helicity may be responsible for the general increase in bacterial activity however, a balance between helicity and amphipathicity could be contributing to the bacterial selectivity of A (A1R, A8R) towards *E. coli* and of A (A8R) towards *S. aureus*. All analogues had lower α-helical content compared to the parent peptide which may account for the observed decrease in toxicity. Interestingly, unlike A (I17K) that showed no improvement in antimicrobial activity, substitution of I17 with R (A (I17R) did improve its activity towards *E. coli* to the same extent as A (A8R). This indicated that individual residues and not just disruption of amphipathicity affects activity against different bacteria in different ways. This again suggests that the different side groups of amino acids may play specific and important roles in AMP activity and interaction with bacterial membranes. These specific interactions may also mitigate any change in activity due to changes in other parameters that contribute to AMP activity.

Another study which modified an AMP sequence with the aim of creating a more regular amphipathic profile showed that the increased amphipathicity resulted in increased activity but also increased toxicity. Interchanging the first two residues of SB056-lin resulted in β-SB056-lin, also known as lin-SB056-1 (Manzo et al., 2015). This modified peptide was reported to have a perfect β-stranded amphiphilic structure. This structural change significantly decreased its MIC when tested against *E. coli*, *P. aeruginosa*, *S. aureus* and *E. faecalis*. This also led to increased haemolytic activity, although overall minimal haemolysis was observed at concentrations close to its MIC. Conversely, comparison of synthetic poly (vinyl ether) block and random copolymers, made of hydrophobic isobutyl vinyl ether and cationic amine vinyl ether, indicated that the more regulated amphiphilic profile of the block copolymer resulted in similar bactericidal activity to the random copolymer. However, it did exhibit lower haemolytic activity due to selectivity for bacterial membranes over erythrocyte membranes (Oda et al., 2011).

More recently, ring-opening polymerisation (ROP) of Phe and Lys N-carboxyanhydrides (NCA) was used to create amphiphilic diblock copolymers which also showed lower MICs and haemolytic activity compared to their random copolypeptide equivalents (Zhou et al., 2010; Su et al., 2017). This study suggested that the improved activity was due to the long section of hydrophobic residues present in the diblock copolymers which were able to insert into the membrane more effectively. It must be noted though that the most active random copolymer contained 10 Lys-NCA and 15 Phe-NCA building blocks whilst the diblock copolymers contained 30 Lys-NCA and 15, 30 or 45 Phe-NCA building blocks. Therefore, increased hydrophobicity and charge rather than regulated amphipathicity may have influenced the activity of these AMP polymers.

Unlike the previous examples, a non-amphipathic, charge-clustered AMP showed quite different results when compared to increasingly amphipathic versions of itself (Stone et al., 2019). The charge clustered AMP 6K-F17 exhibited significantly better antimicrobial activity, MIC 1.6 µM towards *E. coli*, than its most amphipathic counterpart, 1KAMP which had an MIC of 12.5 µM. Additionally, at 40 µM they both showed 0% haemolysis. However, in another set of synthesised peptides where 4 alanines were substituted with 4 leucines to increase overall hydrophobicity, 6K-F17-4L and 1KAMP-4L were created and the charge clustered 6K-F17-4L exhibited lower antimicrobial activity but was also less haemolytic (33% haemolysis at 40 µM) than the more amphipathic analogues. The general trend indicated that in this peptide series with overall higher average hydrophobicity, the more amphipathic the peptide the better its antimicrobial activity. However, even though 1KAMP-4L exhibited strong antimicrobial activity towards *E. coli* (MIC 1.6 µM), it caused 81% haemolysis at 40 μM, making it a weaker therapeutic than the less hydrophobic 6K-F17. Of note was the ability of these AMPs to disrupt bacterial and mammalian lipid vesicles. Both charge clustered AMPs, 6K-F17 and 6K-F17-4L, caused more damage to bacterial lipid vesicles than their respective amphipathic counterparts, 1KAMP and 1KAMP-4L. Conversely, for the Leu substituted AMPs, 6K-F17-4L caused the least disruption to liposomes mimicking mammalian membranes. Similar to the amphiphilic diblock copolymers, it may be that the long section of hydrophobic residues present in these charge clustered AMPs allows for better bacterial membrane insertion and therefore disruption. It was also suggested the due to the charge cluster, it is only after an electrostatic interaction occurs with an anionic membrane that the hydrophobic domain causes membrane disruption. This may also explain the reduced haemolysis observed in 6K-F17-4L compared to 1KAMP-4L. The charge clustered variants were also more resistant to proteolytic degradation. After 60 min incubation with proteinase K, ∼ 60% of 6K-F17 still remained compared to <10% for other peptide variants with a lower charge cluster.

Amphipathicity is a key feature of natural AMPs, but it is unclear whether a more regulated amphipathic profile is always desired in AMP design. In some cases, it can improve antibacterial activity whilst in others, it decreases activity, and the same inconsistency is seen in haemolytic activity. The length of the polar and non-polar sections within an amphipathic AMP may possibly account for this variation. For high antimicrobial activity the hydrophobic sections may need to be a certain length to cause significant membrane damage whilst longer sections of charged residues may improve selectivity for bacterial membranes over mammalian membranes. It is difficult to determine the exact influence of amphipathicity on antibacterial activity as changing amphipathicity also result in changes to helicity, hydrophobicity, and overall positive charge. Each of these parameters are important to AMP activity and are not independent of each other, thus making it difficult to pinpoint precisely how natural AMP features can be optimised through a rational evidence-based strategy, rather than a semi-rational trial and error approach.

#### Overall Charge

In addition to observing the effects of changing amphipathicity, the AR-23 analogues also allowed for analysis of the correlation between overall positive charge and AMP activity and selectivity (Zhang et al., 2016). As alanine was being replaced by the positively charged residues Arg and Lys, the overall net charge of the analogues therefore increased, ranging from +4 for AR-23 to +7 for analogues with 3 substitutions. An increase in charge was well correlated with reduced haemolytic activity. In analogues where I17 was replaced with Arg or Lys, the Arg substitution was more haemolytic than the Lys substitution but nonetheless remained much less toxic than analogues with a lower overall net positive charge. Arg interacts with lipid membranes more strongly than Lys and is further discussed in Section 4.1. As for antibacterial activity, there was no clear correlation with charge and activity. As previously discussed, this may be more a result of the substitutions affecting amphipathicity, helicity and hydrophobicity than a reflection on the effects of increased charge.

An example of the effects of increased charge whilst maintaining amphipathicity, hydrophobicity and length was shown through an analogue of aurein 1.2 (Ramezanzadeh et al., 2021) Substituting Asp 4 and Glu 11 of aurein 1.2 with Lys resulted in the peptide named aurein M2. These substitutions increased the net positive charge from +1 to +5. When tested against *S. aureus*, aurein M2 had an MIC of 12.5 μg/ml which was two-fold better than aurein 1.2 (25 μg/ml), but against *E. faecalis*, the MIC values were the same (50 μg/ml). However, M2 exhibited improved antibacterial activity towards two Gram-negative strains, *E. coli* (MIC of 25 μg/ml) and *P. aeruginosa* (MIC of 6.25 μg/ml), whilst the MIC of aurein 1.2 was >200 μg/ml for both strains. Although M2 was the most helical (78.44% α-helix compared to 53.04% for aurein 1.2), another analogue which in addition to the substituted Lys also substituted Ala 10 for tryptophan (aurein M3), had a lower α-helical content (36.36%) but even better antimicrobial activity. The MIC values for aurein M3 were 3.12 μg/ml (*S. aureus*), 25 μg/ml (*E. faecalis*), 6.25 μg/ml (*E coli*) and 3.12 μg/ml (*P. aeruginosa*). This suggests that the increased charge rather than increased α-helicity of M2 may be responsible for the increase in activity and the addition of tryptophan (Trp) further improves activity despite an even lower α-helical content. Overall, M2 was also the least haemolytic with 5% haemolysis of human red blood cells being caused by 100 μg/ml of M2 compared to 50 μg/ml and 25 μg/ml for aurein 1.2 and aurein M3 respectively.

Similarly, the overall net charge of the 26-residue V13Kʟ peptide was altered by varying the number of positively charged residues but only on the polar face to determine how charge affects activity, while maintaining the non-polar face unchanged (Jiang et al., 2008). Decreasing the charge from +7 to +4 significantly reduced both its antimicrobial and haemolytic activity. However, increasing the charge to +8 increased its activity towards some bacteria whilst maintaining the same haemolytic activity as V13Kʟ. Further increasing the charge continued to improve activity, although it also made the peptide significantly more haemolytic.

Generally, an increase in net charge results in increased activity, but only to a certain extent. Studies in which the net charge of various AMPs have been altered show that once the net positive charge reaches a certain point, no further increase in antibacterial activity is observed although haemolytic activity does increase) (Dathe et al., 2001; Jiang et al., 2008). The turning point at which activity decreases with increasing charge varies for different AMPs with an overall positive charge of +8 or +9 often considered the maximum charge that offers increased activity without significantly increasing the toxicity of the peptide (Zelezetsky and Tossi, 2006).

Altering the structure, hydrophobicity, amphipathicity and net charge of AMPs can have either positive or negative results, indicating that a balance between these different factors is crucial to AMP design and function. It may be reasonable rationalise that perfect helicity, amphipathicity and increased net charge will dramatically improve the antimicrobial activity of *de novo* AMPs as they are defining features of natural AMPs. However, such perfectly structured and highly charged AMPs are not found in nature and synthetic AMPs where these parameters have been improved often display high levels of toxicity. Over the years there has been a lot of effort put into better understanding these common characteristics of AMPs and although considerable progress has been made, it has indicated that a fine balance between them is needed for improving and targeting activity. Additionally, despite their main mode of action being membrane disruption, there are certainly differences observed in their activity towards different species of bacteria. Although outside the scope of this review, a comprehensive analysis of differences between bacterial membranes and correlations between AMP activity and bacterial membrane composition would greatly further our understanding of AMPs and the process of rational design. Effects of changing membrane composition on antimicrobial activity has been explored to some extent. For example, the degree of membrane insertion of aurein 2.2 and aurein 2.3 varied between model membranes composed of different lipid combinations (Cheng et al., 2011). Furthermore, a change in membrane fatty acid composition in *S. aureus* was shown to impact the activity of daptomycin and its ability to form pores (Boudjemaa et al., 2018). Bacterial membranes are constantly changing and are affected by their external environment (Sohlenkamp and Geiger, 2015; Siliakus et al., 2017). A better understanding of the specific interactions between AMPs and bacterial membranes may therefore not only assist in designing AMPs with increased antimicrobial activity but may also allow for better cell specificity.

#### Limitations *In Vivo*

Whilst there has been some success in using AMPs topically, systemic therapy has been limited for several reasons (Costa et al., 2019). AMPs are disadvantageous from a therapeutic standpoint as they are susceptible to proteolytic degradation, less active under physiological conditions due to the presence of serum salts and proteins, have high MICs, low selectivity for pathogens over mammalian cells, are toxic to mammalian cells and have a short half-life *in vivo* (Drayton et al., 2020; Hancock and Sahl, 2006; Liu et al., 2010). These limitations are progressively being addressed as AMPs are modified to improve their stability, activity, and biocompatibility (Table 1).

**TABLE 1:** Summary of how different modifications can increase or decrease the antimicrobial activity and toxicity of AMPs.

Modification	Unmodified				Modified^a^				Ref
	Name	MIC		Haemolytic activity^b^	Name	MIC		Haemolytic activity^b^	
		*S. aureus*	*E. coli*			*S. aureus*	*E. coli*		
Increase in amphipathicity due to substitution on polar face	AR-23, 4 + charge	6.25 µM	25 µM	3.13 µM = 37.9%	A (A8R), 5 + charge	3.13 µM	12.5 µM	3.13 µM = 12.9%	Zhang et al. (2016)
Decrease in amphipathicity due to substitution on non-polar face					A (I17K), 5 + charge	50 µM	25 µM	100 µM = 33%	Zhang et al. (2016)
replacement of all Lys with Arg	BP100	32 µM	2 µM	>100 µM = 50%	R-BP100	6 µM	0.9 µM	50.9 µM = 50%	Torcato et al. (2013)
using d-amino acids instead of l-amino acids	L form KLKLLLLLKLK-NH2 (L5)	16 μg/ml	16 μg/ml	N/A	D form klklllllklk-NH2 (DL5)	1 μg/ml	8 μg/ml	N/A	Manabe and Kawasaki, (2017)
	L form Temporin A	8 μg/ml	N/A	N/A	D form Temporin A	8 μg/ml	N/A	N/A	Manabe and Kawasaki, (2017)
Peptoid mimic of a peptide	GN-4 peptide	6.25 μg/ml	6.25 μg/ml	100 μg/ml = 10%	GN-4 peptoid	32 μg/ml	64 μg/ml	>128 μg/ml = 10%	Mojsoska et al. (2015)
addition of a positive charge at the N-terminus	W6-Hy-a1	8 µM	32 µM	4 µM = 50%	K0-W6-Hy-a1	4 µM	4 µM	4 µM = 50%	Crusca et al. (2011)
increased peptide length from 19 residues to 26, but same overall charge	KIA19, +7 charge	>256 μg/ml	64 μg/ml	512 μg/ml = 10%	KIA (7)26	8 μg/ml	4 μg/ml	8 μg/ml = 39% 32 μg/ml = 72%	Gagnon et al. (2017)
dimerisation of magainin 2 at C-terminal	MG2	128 µM	16 µM	32 µM = 0%	(MG2)2K	16 µM	1 µM	32 µM = 60%	(Lorenzón et al., 2016)
dimerisation of magainin 2 at N-terminal	MG2	128 µM	16 µM	32 µM = 0%	E (MG2)2	128 µM	16 µM	32 µM = 5%	Lorenzón et al. (2016)
multimerising the AMP R4 (RLYR) via attachment onto a dendrimeric core	(R4)4	1.8 µM	1 µM	338 µM = 50%	D4 (R4)	0.8 µM	0.6 µM	1,510 µM = 50%	Tam et al. (2002)
change in architecture from linear to star-shaped	L-Lys	16 μg/ml	64 μg/ml	N/A	S-Lys	16–32 μg/ml	32 μg/ml	>10,000 μg/ml = 50%	Wong et al. (2016)
change in architecture from linear to star-shaped	Lys-Val linear polymer	213.37 µM (MBC)	29.5 µM (MBC)	674.5 µM = 50%	S16	4.58 µM (MBC)	0.72 µM (MBC)	58.3 µM = 50%	Lam et al. (2016a)
increasing arm length from medium to very long	SNAPP S4_M_	N/A	2.636 µM	14.727 µM = IC_50_	S4_VL_	N/A	0.403 µM	2.531 µM = IC_50_	Shirbin et al. (2018)
increasing arm number from 4 to 16	SNAPP S4_M_	N/A	2.636 µM	14.727 µM = IC_50_	S16_M_	N/A	0.127 µM	1.090 µM = IC_50_	Shirbin et al. (2018)

a For modified compounds as compared to unmodified, green highlighted sections indicate improved activity, red highlighted sections reduced activity and yellow highlighted sections indicating no change.

b Haemolytic activity = concentration of compound that causes x % haemolysis expressed as µM unless otherwise indicated.

## Optimising Linear Antimicrobial Peptides

### Type and Sequence Position of Amino Acids Enhance Antimicrobial Activity of Antimicrobial Peptides

Engineered AMPs that are high in hydrophobic and basic amino acids have been shown to have improved antimicrobial activity (Wimley, 2010). Identification of specific amino acid residues that contribute to improvement has been aided by using the technique of alanine scanning (sequential substitution of each peptide residue with Ala) of naturally occurring AMPs. When analogues of aurein 1.2 were synthesised by substituting each amino acid for Ala one at a time, it was only the replacement of aspartic acid (Asp), glutamic acid (Glu) and serine (Ser) at positions 4, 11 and 12, respectively, that resulted in improved activity (Migoń et al., 2019). All other substitutions reduced activity when compared to native aurein 1.2. These substitutions highlighted how hydrophobicity, resistance to proteolytic degradation and helical structure are affected by different residues. Replacement of Asp and Glu improved antimicrobial activity, but their removal also resulted in lower proteolytic stability and reduced α-helicity. The increase in activity was therefore best explained by their removal resulting in an increase in the overall positive charge of the analogue. The increase in activity observed by substituting Ser12 with Ala was contributed to the resulting increase in hydrophobicity and stability. However, the Ser12 analogue was less active towards *P. aeruginosa*, indicating that Ser may be important for interacting with the bacterial membrane. Phenylalanine 3 and 13 had previously been noted as being important for membrane anchoring, and their contribution to aurein 1.2 activity was further demonstrated in this study as substitution with Ala resulted in decreased activity (Shahmiri et al., 2017). Varying the position of Lys within the sequence also produced very different results, despite both the Lys being located next to each other at positions 7 and 8. Both K7A and K8A exhibited reduced activity compared to aurein 1.2, yet K8A had a lower MIC than K7A when tested against *E. faecalis*, *E. coli* and *Candida albicans*. K8A was also more haemolytic and had a higher α-helical content in dodecylphosphocholine (mammalian membrane mimic) but it had lower α-helical content in sodium dodecyl sulfate (bacterial membrane mimic) and was less stable than K7A.

Other *in vitro* studies have illustrated that Trp can improve the antimicrobial activity of AMPs and increase their resistance to proteolytic degradation (Deslouches et al., 2005; Hilpert et al., 2005; Shang et al., 2016; Han et al., 2020). It was also noted that Trp is particularly suited for targeting Gram-negative bacteria, as it can strongly bind to the LPS that make up the outer membrane. The Trp residues are thought to help dissociate the LPS of Gram-negative bacteria, allowing the peptide to pass the outer membrane and then disrupt the cytoplasmic membrane. Within the overall context of a peptide, these studies showed that the addition of Trp was more effective when it was located at the amino terminus compared to the carboxyl terminus (Bi et al., 2013; Shang et al., 2016).

Arg and Lys are also regularly used in AMP design. However, despite both residues having the same charge, substitution of Lys with Arg can further improve activity (Torcato et al., 2013). This was shown by replacing all Lys of the synthetic AMP BP100 with Arg to create R-BP100. This substitution resulted in a twofold decrease of its MIC when tested against *E. coli* and a fivefold decrease against *S. aureus*. This increase in activity is possibly due to the side chain of Arg containing a guanidino group which can interact with two lipid headgroups, whereas the side chain of Lys can only bind one (Rothbard et al., 2005). The amine side chain of Lys is also known to become deprotonated in a lipid membrane. This leads to weaker interactions with water and lipid headgroups and does not cause extensive damage to the membrane. Arg, however, maintains its positive charge in the membrane environment and its ability to form more H-bonds with lipid head groups leads to membrane thinning and pore formation (Li et al., 2013). With certain residues exhibiting the ability to improve AMP activity, peptides enriched in these residues have been developed as another means to create optimised compounds.

The occurrence of natural AMPs enriched in certain residues has led to the development of synthetic AMPs also enriched with certain residues, to further increase their charge and hydrophobicity and improve activity (Chan et al., 2006; Stensvåg et al., 2008). For example, substituting different residues of the neuropeptide α-melanocyte stimulating hormone (α-MSH (6–13)) with Arg and Trp created different combinations of Arg/Trp rich peptides with enhanced charge and hydrophobicity (Singh et al., 2020). The most charged and hydrophobic peptide from this group (Ana-5) exhibited improved antibacterial activity and serum stability compared to its parent peptide. Similarly, analogues of a truncated aurein 2.2-∆3 enriched with Arg and Trp exhibited improved activity but were also more cytotoxic (Kumar et al., 2017, 2019; Raheem et al., 2020). Replacing neutral and non-polar residues on the polar face of the α-helical AMP Temporin-1CEb with Lys is another example of an AMP that was modified to decrease haemolytic activity and increase antimicrobial activity (Shang et al., 2012). This resulted in a series of Lys-rich AMPs whereby replacement with six lysine (analogue named *L*-K6) resulted in high antimicrobial activity and low haemolysis. This Lys-rich analogue was also shown to be able to neutralise LPS and prevent it from inducing a proinflammatory response (Dong et al., 2018).

Proline (Pro) rich AMPs (PrAMPs) are another group of antimicrobials, predominantly found in insects, that translocate across the bacterial membrane to act on internal targets and can modulate the immune system (Otvos and Otvos, 2002; Li et al., 2014). Based on naturally occurring pyrrhocoricin, drosocin and apidaecin, a series of PrAMPs was created and then further optimised by creating C-terminally linked dimeric structures and adding an amino-cyclohexyl carboxylic acid moiety (Chex) to the N-terminal (Otvos et al., 2005; Otvos et al., 2014). From this series, A3-APO was identified as the best peptide and showed considerably better activity towards a range of fluroquinolone-resistant *E. coli* and *K. pneumoniae*, compared to the clinically used antibiotic ciprofloxacin. Reviews by Welch et al. and Li et al. provide a more in depth look at this class of AMPs (Li et al., 2014; Welch et al., 2020).

### Side-Chain Length, d-amino Acids and N-Terminal Modification

Altering the side-chain length of AMP residues is another way in which activity can be manipulated. With regards to activity against *P. aeruginosa*, peptide/α-peptoid oligomers with shorter cationic side-chain lengths resulted in a more hydrophobic surface and improved membrane disruption. This increased hydrophobicity is believed to be due to the aromatics in the oligomers shielding the cationic side chains, since longer cationic side chains resulted in reduced hydrophobicity. As previously noted in other AMPs, an increase in hydrophobicity also correlated with an increase in haemolysis and cytotoxicity (Frederiksen et al., 2019). Using D-amino acids instead of ʟ-amino acids can also sometimes improve activity. The D-enantiomer of a sapecin B analogue, named DL5, was compared to its ʟ-isomer and was found to have a much lower MIC value when tested against *S. aureus* and *E. coli*. Of note, its activity towards *S. aureus* was improved by 16-fold compared to only a two-fold increase in activity towards *E. coli*. This is potentially due to its higher affinity for *S. aureus* peptidoglycan than the ʟ-form (Manabe and Kawasaki, 2017). However, using D-amino acids does not always improve activity. The D-form and ʟ-forms of Kn2-7, Mastoparan M and Temporin A were also tested but their D-forms did not show improved activity.

Another study compared the ʟ-enantiomers of V681, V13Kʟ, and V13AD with their corresponding D-isomers (D-V681, D-V13KD, and D-V13Aʟ) (Chen et al., 2006). Again, it was observed that, generally, D-isomers did not display significantly different antimicrobial or haemolytic activity compared to their ʟ-isomer counterparts. Nevertheless, they did show significantly improved resistance to proteolytic degradation. The peptides were incubated with trypsin at a 20,000:1 (peptide: trypsin) ratio for 8 h at 37°C. After 60 min, all ʟ-peptides were fully degraded as determined by RP-HPLC. On the other hand, all D-peptides were fully intact even after 8 h incubation. The peptide D-V13KD stood out with the highest therapeutic index (TI). Its antibacterial activity in physiological conditions was further investigated along with its ʟ-isomer (Huang et al., 2011). Again, both isomers had similar haemolytic activity and MIC values when tested against human red blood cells and Gram-negative bacteria. However, D-V13KD had lower MIC values towards Gram-positive bacteria compared to V13Kʟ. The MIC of these peptides towards *E. coli*, *P. aeruginosa*, *S. epidermidis* and *B. subtilis* was also evaluated when in the presence of either NaCl (150 mM), CaCl2 (2.5 mM) or human serum albumin (HSA, 0.6 mM). For both peptides, their antibacterial activity was decreased when tested in these mediums, but the decrease in activity of D-V13KD was significantly less than for V13Kʟ.

N-terminal modification is another method for improving AMP activity. Analogues of Hy-al, an AMP isolated from the South American frog *Hypsiboas albopunctatus,* were made via solid phase peptide synthesis (SPPS) that had either an acetyl group, Lys or Asp added to the N-terminus (Crusca et al., 2011). These additions resulted in analogues with N-terminal no charge (Ac^0^-W^6^-Hy-a1), positive charge (K^0^-W^6^-Hy-a1) or negative charge (D^0^-W^6^-Hy-a1) respectively. At position 6, Leu was also substituted with Trp, and an unmodified-N-terminus analogue made (W^6^-Hy-a1). The addition of Trp did not change the MIC compared to unmodified Hy-a1 but did significantly increase its haemolytic activity. This increase in haemolytic activity was observed for all N-terminally modified analogues, although K^0^-W^6^-Hy-a1 also exhibited increased antimicrobial activity against *E. coli*, *S. aureus*, *P. aeruginosa* and *B. subtilis*, thus increasing the TI of this analogue.

Some other N-terminal modifications that lead to improved activity include the addition of acyl groups or fatty acid chains. Amino acid residues 21–31 of lactoferrin were synthesised to create LF11, which exhibited weak antibacterial activity (Zweytick et al., 2011). In one example, the authors sought to improve the activity of LF11 by exchanging Lys 9 and Val 10 for Ile and Arg respectively and deleting Gln 2 and 4 and asparagine (Asn) 6 which resulted in LF11-215. They then added either octanoyl (O-LF11-215) or 6-methyl-octanoyl (6-MO-LF11-215) to the N-terminus of this optimised analogue. This additionally reduced the MIC of LF11-215 from 16 to 32 μg/ml to 10 μg/ml for both analogues when tested against *E. coli*. Other studies have added different length fatty acid chains to AMPs and have found that this addition increases overall hydrophobicity, resulting in improved activity. One study added different length alkyl chains to the LL-37 derived peptide KR12. They found that increasing alkyl chain length up to 8 carbons (C_8_) improved activity but beyond that a decrease in activity was observed despite compounds with longer chains being more hydrophobic (Kamysz et al., 2020). This was possibly due to compounds with longer chains being more prone to self-assembly. Addition of fatty acid chains also increased haemolytic activity but C_8_-KR12-NH_2_ showed <10% haemolysis at concentrations well above its MIC. However, addition of a longer chain, C_10_, significantly increased the haemolytic activity. In another study, adding fatty acid chains of increasing length to an anoplin analogue (anoplin-D4, 7) also resulted in improved activity and increased haemolysis that correlated to chain length (though haemolysis was again only 10% at concentrations above the MIC values of C_10_-D4, 7 and C_12_-D4, 7, and C_8_-D4, 7 was not haemolytic) (Zhong et al., 2019). The effect of lipidation on AMP activity is further reviewed by Rounds and Straus (2020) and Li et al. (2021).

### Optimal Peptide Length

Overall charge affects AMP activity, making it difficult to determine the effect of peptide length. One study overcame this by building on repeats of KIAGKIA (the repeating unit of the peptide MSI-103, to create α-helical analogues of different lengths but with a constant charge of 7^+^ by substituting certain Lys for Ala (KIA peptides) or Lys for Ser (KISA peptides) in subsequently longer analogues (Gagnon et al., 2017). This study showed that independent of charge, longer peptides were generally more active than their shorter counterparts and that a minimal length of ∼17–19 residues was required for membrane disruption of *E. coli*, *P. aeruginosa* and *S. aureus*. Activity increased with increasing length, but this came at a cost. Peptides ≤17–19 residues long showed no haemolysis (or activity), but peptides ≥21 residues long showed high haemolysis at varying concentrations. Interestingly, this study also observed that in some cases the longer peptide was slightly less active than the previous shorter peptide in their series. It was noted that these longer, less active peptides contained Lys at the C-terminus, whereas the next shorter, more active peptides contained the hydrophobic residues Ile and Ala. This suggests that a more hydrophobic C-terminus may aid in membrane insertion and can result in a shorter peptide being more active than a longer peptide. The peptides were also tested in vesicle leakage experiments using lipid vesicles of different membrane thickness. Short peptides (<17 residues), that were inactive towards bacteria, could cause leakage in vesicles with thinner membranes but were unable to in vesicles with thicker membranes. A clear correlation between peptide length and membrane thickness was observed. This indicated that for this series of peptides, they needed to be able to span the bacterial membrane in order to exert activity. As such, optimal peptide length would depend on the bacteria it is being tested against and the thickness of its membrane.

Short, 6 residues peptides have nonetheless shown to be active against bacteria, suggesting that not all AMPs need to be able to span the membrane to exert activity. The Arg/Trp-rich nature of these short peptides also contribute to their activity, making their activity as a function of length incomparable to the activity of the KIA peptide series (Strøm et al., 2002). Ultrashort 3 residues long Arg/Trp-rich peptides are another inactive peptide, although when they are multimerised onto divalent and trivalent benzene scaffolds, the resulting compounds have higher activity that show negligible haemolysis, revealing multimerisation as another tool for improving AMPs (Hoffknecht et al., 2015).

## Multi-Unit Materials With Antimicrobial Activity

### Multimeric Antimicrobial Peptides

**FIGURE 3:**
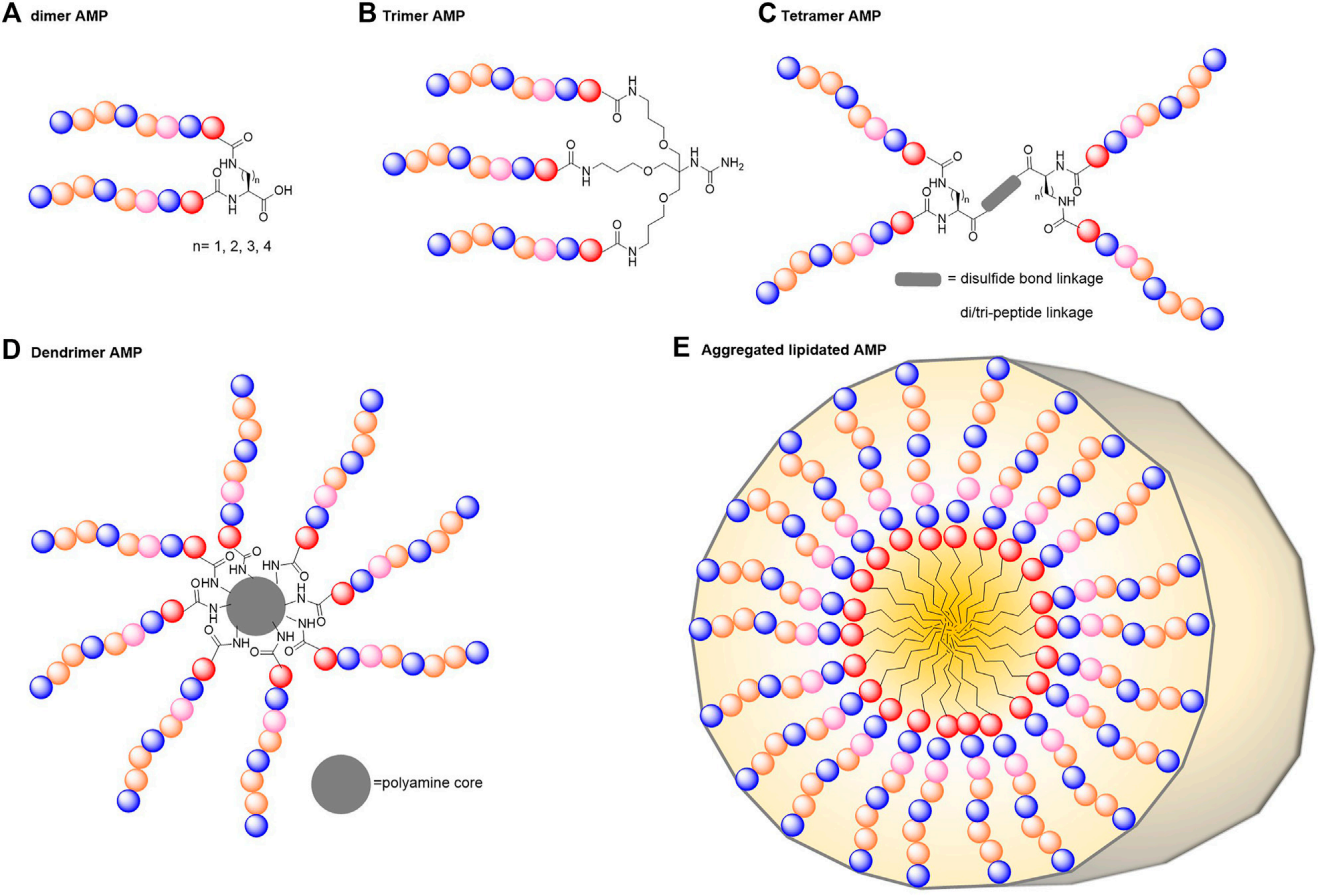
Possible architectures of multimerised peptides. AMPs can be multimerised into various forms to improve their activity and biocompatibility. These include dimers **(A)**, trimers **(B)**, tetramers **(C)**, larger multi-armed dendrimers **(D)** or self-assembled peptide multimers **(E)**.

Synthetic AMPs made of repeat units and constructed into branching forms can help resolve some of the limitations currently faced by linear AMPs (Figure 3). Multimerisation has been shown to increase AMP ability to disrupt bacterial membranes as well as their stability in physiological conditions to such an extent that any increase in cytotoxicity and haemolytic activity is negligible (Wiradharma et al., 2012; Li et al., 2015). These multimeric constructs can be made in various ways such as polymerisation, intermolecular covalent linking, multigeneration dendrimers or attachment to unnatural scaffolds or core molecules.

#### Peptide Based Antimicrobial Polymers

AMP-based polymerisation has also been employed to help overcome some AMP limitations. Anionic ring-opening polymerisation (ROP) was used to create poly-β-peptides, around 20 residues in length, with un-natural β-amino acids. The final structure of these compounds contained hydrophobic and cationic subunits (Zhang et al., 2019). Unlike standard AMPs, this polymer did not have a defined secondary structure but showed considerable activity towards a variety of Gram-positive and Gram-negative bacteria, low haemolytic activity and negligible cytotoxicity. Its MIC values were 12.5 μg/ml, 25–50 μg/ml, and 3.13–12.5 μg/ml for various strains of *S. aureus*, *E. coli* and *P. aeruginosa,* respectively. A 20:80 ratio of hydrophobic to cationic subunits showed the best activity, with more than 20% hydrophobicity leading to increased toxicity whilst less than 20% reduced its activity (Chen A. et al., 2020). N^ε^-tert-butyloxycarbonyl-D,l-lysine (Boc-DLL) and γ-benzyl-l-glutamate (BLG) polymerised in a 90:10 ratio also exhibited high activity against various clinical strains of multi-drug resistant (MDR) *P. aeruginosa* with an MIC of 50 μg/ml (Jiang et al., 2020). Extremely low haemolytic activity and no cytotoxicity at its MIC was observed. Other interesting polymeric compounds are poly (ester amide) (PEA) random copolymers made from Lys, Arg and Phe (Zhu J. et al., 2019). The amphiphilic profile of this polymer allowed it to form micelles in water with the hydrophobic Phe protected by Lys and Arg. MIC values were not calculated but the OD growth curves of *E. coli* and *S. aureus* increased more slowly in the presence of PEA micelles. SEM images of treated and untreated bacteria also suggested a membrane lytic mode of action. Haemolysis and cytotoxicity were observed at concentrations of 500 μg/ml and 2 mg/ml, respectively, and displayed no significant toxicity. A wound infection mouse model also showed that treatment with PEA micelles reduced both bacteria load and the amount of various inflammatory cytokines in the wound tissue and serum. AMP activity was further increased by conjugation of the antibiotic levofloxacin (LV) or by preloading the micelles with LV, showing that they could also be used as nanocarriers for drug delivery.

#### Dimeric and Branched Antimicrobial Peptides

Dimeric AMPs (Figure 3A) exist in nature and exhibit certain antimicrobial advantages over linear forms. The naturally occurring AMP distinctin has a unique structure of two independent peptide chains (chain A and chain B) that form a heterodimer via a disulfide bond (formed between the C-terminal cysteines) (Batista et al., 2001). Analogues of this natural dimer, either linear forms of just chain A or B as well as A-A and B-B homodimers, were compared to distinctin and demonstrated how different structures affect activity and biocompatibility (Dalla Serra et al., 2008). The analogues were highly susceptible to protease degradation by elastase, whereas distinctin exhibited good stability. When tested against a variety of Gram-positive and Gram-negative bacteria, the homodimers had similar MIC values compared to distinctin and were lower than their linear counterparts, but both the heterodimers and homodimers were marginally more cytotoxic than the linear A and B chains.

The presence of such dimers in nature has led to synthetic AMP dimers being created, although with varying results (Liu et al., 2010). Dimerisation of magainin 2 was achieved either by replacing the C-terminal asparagine with a cysteine and forming a disulfide bond or by using Lys at the C-terminus or Glu at the N-terminus as ligands for the two chains (Dempsey et al., 2003; Lorenzón et al., 2016). N-terminal dimerisation did not improve the activity of magainin 2, however, C-terminal dimerisation using Lys or a disulfide linkage significantly improved its activity, albeit at the cost of increased haemolysis. Conversely, dimerisation of aurein 1.2 at both the C-terminal and N-terminal showed decreased activity compared to its linear form but had minimal haemolytic activity (Lorenzón et al., 2013). N-terminal dimerisation of PST13-RK also exhibited decreased activity and increased cytotoxicity compared to its linear form (Yang et al., 2009).

While acknowledging that dimerisation does not always lead to AMP improvement, by expanding on the concept that multiples of a peptide can improve overall activity, leads us to branched AMPs (Figures 3B,C). Branched AMPs can be formed through several methods and show improved activity. Comparison of dimeric (K-2A), trimeric (2K-3A), and tetrameric (3K-4A) anoplin, which were additionally lipidated at the N-terminal, revealed the trimer with *n*-butyric acid (2K-3A-C4) to have the best antibacterial activity (Gou et al., 2021). All of the multimers were built from a branched lysine core and tested against *S. aureus*, *B. subtilis*, *E. coli*, *P. aeruginosa* and *K. pneumoniae.* Overall, the trimer was found to be the most active followed by the dimer, and then the tetramer with all multimers more potent than linear anoplin-C4. This showed that like many other modification strategies, there is an optimal state for each compound whereby optimisation past a certain point does not further improve activity. Unfortunately, these multimers also showed increasing haemolytic activity and cytotoxicity with increasing multimerisation. Another example of a branched AMP is the optimised proline-rich AMP (Chex1-Arg20) that used 2,4-diaminobutyric acid (Dab) to form a C-terminally linked homodimer (A3-APO) (Otvos et al., 2005). APO tetramers were then formed from this homodimer by either further building upon small molecule scaffolds or by adding a cysteine to its C-terminus and forming a disulfide bridge between two A3-APO molecules (Otvos et al., 2014; Li et al., 2015). It was shown that in the case of Chex-Arg20, the MIC and minimum bactericidal concentration (MBC) values of the dimer and tetramer were not significantly lower than the monomer. However, their ability to permeabilise *E. coli* membrane was increased through dimerisation with the tetramer further showing much stronger activity at lower concentrations. It can therefore be seen how, in some cases, further multimerisation provides an alternative strategy to increase activity and can change the mode of action of an AMP.

#### Dendrimeric Antimicrobial Peptides

Multimerising AMPs by tethering them onto dendrimers is another method for creating improved antimicrobial molecules (Figure 3D). Lysine is often used to create dendrimeric cores as it is a facile method for creating multivalent structures (known as multiple antigen peptides (MAPs)). Early work by Tam et al*.* showed that AMPs with low activity in their monomeric forms display potent activity when incorporated into tetra- and octavalent dendrimers (Tam et al., 2002).

Linear compounds of RLYR (R4) and two, four and eight repeats thereof were synthesised ((R4)_2_ (R4)_4_, and (R4)_8_ respectively), and their antimicrobial activity was compared to divalent, tetravalent and octavalent lysine-core dendrimers (D_2_, D_4_ and D_8_, respectively) which were decorated with R4 peptide to form the Lys-dendrimer-R4 constructs D_2_R4, D_4_R4 and D_8_R4 respectively (Tam et al., 2002). A longer R8 peptide attached to a single Lys (RLYRKVYG(K)) was also synthesised as well as its divalent, tetravalent and octavalent analogues (D_2_R8, D_4_R8, D_8_R8 respectively). Antimicrobial activity assays were conducted in both low and high salt conditions (as a simulation of physiological conditions) for a variety of Gram-negative and Gram-positive bacteria as well as some fungi. The monomeric R4 and R8 peptides showed no and low activity respectively in low salt conditions and R8 activity was completely abolished in high salt concentrations. The divalent dendrimers of each group showed better activity than their linear counterparts under low salt concentrations, but this was also diminished when tested in high salt. Notably, D_4_R4 and D_4_R8 not only showed improved activity compared to the monomers and divalent dendrimers (e.g., low salt MIC values of >500 µM for R4 and 6.1 µM for D_2_R4 compared to 0.6 µM for D_4_R4 against *E. coli*), but also retained their activity at high salt concentrations (with only a very slight increase in MIC observed). D_4_R4 was generally slightly more active than D_4_R8 and the octavalent dendrimers of R4 and R8 showed only small improvements in activity. The dendrimers did show increased haemolysis compared to R4 but based on molecular size, the dendrimers consistently showed reduced haemolytic activity (EC_50_ of (R4)_4_ was 338 µM compared to 1,510 µM for D_4_R4). The EC_50_ values for all linear and dendrimeric compounds were, however, well above their respective MIC values and thus considered nontoxic. Their activity after exposure to the proteolytic enzymes trypsin and chymotrypsin was also measured. After 24 h incubation with trypsin, D_4_R4 retained 80% of its activity against *E. coli* whereas (R4)_4_ only retained ∼20% activity, indicating that the branched dendrimeric structure provided improved proteolytic stability.

The tetravalent dendrimer decorated with RW ((RW)_4D_) is another example of a multivalent dendrimer that showed increased activity and a reduced haemolytic index (HI) in physiological salt conditions compared to the naturally occurring Arg/Trp rich AMP indolicidin (Liu et al., 2007b). Its increased activity towards *E. coli* and *S*. *aureus* correlated with increased haemolysis but its HI was much higher than that of indolicidin. Interestingly (RW)_4D_ was more active towards *E. coli* than *S. aureus*, whereas the reverse was observed for a series of linear peptides made from repeating units of RW, suggesting that AMPs multimerised onto a dendrimeric core may preferentially target Gram-negative bacteria (Liu et al., 2007a). Conversely, dendrimerisation of the linear peptide lin-SB056-1 onto a divalent lysine scaffold with an 8-aminooctanamide (Aoc) hydrophobic tail (den-SB056-1) did not improve its antibacterial activity when tested against *E. coli* and *S. aureus* in Mueller Hinton broth (MHB) (Batoni et al., 2016). However, when tested in MHB media in the presence of salts, den-SB056-1 had a much lower MIC than lin-SB056-1 (MIC value of 3.125 and 12.5 µM respectively against *E. coli* and 6.25 µM and >100 µM against *S. aureus*). This indicated that a multimerised peptide form may help AMPs maintain their activity in salt environments. Again, although the dendrimer did display increased haemolysis, at concentrations close to its MIC <20% haemolysis was observed.

Another dendrimer that has shown potent antimicrobial activity is the amphiphilic branched analogue of lysine termed BALY (Lind et al., 2014). The MIC of BALY towards *S. aureus*, MRSA, *E. coli*, and *P. aeruginosa* was 0.93, 10, 12, and 51 μM, respectively. The interaction of BALY with bilayer membranes that were in either a gel-phase or a fluid-phase, was closely studied and membrane thinning was observed in a gel-phase membrane whereas spherical aggregates were observed in fluid phase membranes. Further studies also revealed that BALY only interacted with the outer leaflet of homologous fluid- or gel-phase membranes, but that it caused increased disruption to mixed membranes and could penetrate across the lipid bilayer (Lind et al., 2015). These studies indicate that forming AMP dendrimers can impact their mode of action.

Recent research has also reported the creation of a series of dendrimer-based AMP-like compounds that take on an umbrella architecture and exhibit strong antimicrobial activity and low haemolysis and cytotoxicity (Chen S. et al., 2020). This series is comprised of 2,2-bis(hydroxymethyl) propionic acid (bis-MPA) dendrons of first (G_1_), second (G_2_) and third (G_3_) generation displaying two, four and eight β-alanine groups respectively that form a cationic canopy. This canopy acts as a shield and is bound to hydrophobic alkyl chains varying from two to fourteen carbons long (C_2_-C_14_), creating the umbrella-like structure. The division of the cationic canopy on one side and the hydrophobic tail on the other is reminiscent of the amphiphilic profile of AMPs. When tested against *E. coli* and *S. aureus*, increasing alkyl chain length correlated with an increase in antimicrobial activity. The most active compound was a second generation dendron with an alkyl chain of C_14_ (C_14_G_2_) with MIC values of 3.9 and 1.95 μg/ml for *E. coli* and *S. aureus,* respectively. As previously noted, haemolysis is often associated with the overall hydrophobicity of a compound. When comparing dendrons of the same generation, increasing alkyl chain length, hence overall hydrophobicity, resulted in increasing haemolysis (HC_50_ 1,514, 79.6, and 10.1 μg/ml for C_8_G_1_, C_10_G_1_ and C_14_G_1_ respectively). However, higher generation dendrons altered overall hydrophobicity, as seen by comparison of C_14_G_1_, C_14_G_2_ and C_14_G_3_ which have HC_50_ values of 10.1, 63 and ∼5,000 μg/ml, respectively. C_14_G_3_ was highlighted as the most promising compound and its cytotoxicity towards HeLa cells was considered mild with an LC_50_ of 85 μg/ml. The cytotoxicity of C_2_G_3_ and C_14_G_2_ was also tested and comparison of their LC_50_ values (>250 and 32 μg/ml respectively) with that of C_14_G_3_ showed that again increased tail length correlated with increased toxicity and that higher generation dendrons can mitigate this toxicity to an extent.

#### Star-Shaped Antimicrobials

An extension of branched and dimeric architecture is the collection of molecules known as star-shaped polymers. Their star architecture further confers enhanced activity, biocompatibility and stability compared to their linear counterparts. Arms made of polylysine and polyglucosamine radiating from a cross-linked core form a type of star-shaped polymer in which antimicrobial activity and biocompatibility can be regulated by varying the ratio of polylysine to polyglucosamine (Wong et al., 2016). Various star polymers with an estimated number of arms ranging from 12 to 19 were made via a combination of reversible addition–fragmentation chain-transfer (RAFT) polymerisation, N-carboxyanhydride ring-opening polymerisation (NCA-ROP) and click chemistry. The mol percentage of the glycopolymer polyglucosamine (GSA) arms to polylysine was also adjusted so that star polymers were generated with either 25, 50, 65 or 100 per cent of GSA arms for star molecules with a total of about 13 (S-GSA 25), 19 (S-GSA 50), 13 (S-GSA65) or 12 (S-GSA 100) arms respectively. Linear (L-Lys) and star (S-Lys) molecules containing only polylysine were also constructed. Their activity in MHB showed that the star architecture provided increased activity against *E. coli*, *P. aeruginosa* and *S. aureus* as indicated by their respective MIC values (Table 2). At 100 μg/ml, both compounds were extremely cytotoxic towards human aortic smooth muscle cells (AoSMC) however, all other S-GSA compounds resulted in >80% cell viability (improving with higher GSA content), indicating that the GSA arms were needed for reduced cytotoxicity. A mixture of L-Lys and linear GSA (L-GSA) at a 75:25 M ratio was also tested and shown to be more cytotoxic than S-GSA 25, highlighting that the star architecture also plays a role in improving biocompatibility.

**TABLE 2:** Summary of activity of star-shaped antimicrobials^a^.

Antimicrobial Activity	Compound name^b^	*E. coli*	*P. aeruginosa*	*A. baumannii*	*S. aureus*	HC_50_
MIC (µg/ml)	**L-Lys**	64	256		16	
	**S-Lys**	32	256		16–32	>10,000
	**S-GSA 25**	>512	>512		32–64	>10,000

MBC (µM)	**Lys/Val linear analogue**	29.50			213.37	674.5
	**S16**	0.72	1.42	0.85	4.58	58.3
	**S32**	0.72	0.97	0.79	2.23	45.3
MIC (µM)	**S16**_**M**_ **(14 DP)**	0.127				
	**S4**_**M**_ **(12 DP)**	2.64				
	**S4**_**L**_ **(19 DP)**	0.56				

MIC (µM)	**Linear PLL**		5.3	10.5	2.6 (MRSA)	>4,000
	**P2 (8 arm, 10 DP)**		2.3	4.5	1.1 (MRSA)	>4,000
	**P4 (8 arm, 20DP)**		2.3	2.3	0.6 (MRSA)	>2000
	**P6 (15 arm, 10 DP)**		2.4	2.4	1.2 (MRSA)	>2000

The bold values/text is to highlight the compounds, the meaning of each of these is in the text

a References: (Lam et al., 2016a; Shirbin et al., 2018; Lu et al., 2019).

b Highlighted in orange are the lead compounds for each class of star-shaped antimicrobial.

This is possibly due to this star-structure allowing the GSA arms to shield the polylysine arms from non-specific interactions with other cells. Additionally, none of the star molecules were haemolytic at concentrations of 10,000 μg/ml, which was attributed to the lack of hydrophobic groups. S-GSAs also exhibited preferential activity towards Gram-positive bacteria compared to Gram-negative. It was suggested that the glucosamine shielded the Lys arms from interacting with the outer membrane of Gram-negative bacteria. Conversely, glucosamine, which resembles peptidoglycan structure, was thought to be responsible for penetrating the Gram-positive peptidoglycan layer so that the polylysine could then induce cell death. Unfortunately, the mechanism of action of these molecules was not described. Of all the compounds tested, S-GSA 25 was highlighted as the most promising antimicrobial as it had the highest TI of 7.0 as determined by its MIC against *S. aureus*.

Star-shaped structurally nanoengineered antimicrobial peptide polymers (SNAPPs), created through ROP of Lys and Val NCA building blocks at a theoretical ratio of 2:1 onto an amine core have also been shown to be effective in killing pathogenic bacteria (Lam et al., 2016a; Lam et al., 2016b). Compared to a linear analogue that represented one arm of these star molecules, the star architecture showed improved activity. Both the 16-arm (S16) and 32-arm (S32) SNAPPs had much lower MBCs in MHB compared to the linear analogue against *E. coli* and *S. aureus* (Table 2). These SNAPPs were also highly active against *K. pneumoniae* and *A. baumannii* with MBCs of 1.54 and 0.83 µM (S16 and S32) and 0.85 and 0.79 µM (S16 and S32) for *K. pneumoniae* and *A. baumannii*, respectively. The S16 and S32 SNAPPs exhibited considerably better activity towards these Gram-negative bacteria than towards the Gram-positive bacteria *S. aureus* (MBC of 4.58 and 2.23 µM for S16 and S32 respectively) and *S. mutans* (MBC of 3.55 and 1.80 µM for S16 and S32 respectively). They also showed very low haemolytic activity, with concentrations >100 × MBC resulting in <30% haemolysis and they were compatible with human embryonic kidney cells and rat hepatoma cells (IC_50_ values well above MBC values). When tested *in vivo* in a mouse peritonitis model infected with colistin and multi-drug resistant (CMDR) *Acinetobacter baumannii* (*A. baumannii*), S16 was effective in reducing bacteria in the peritoneal cavity, blood, and spleen. It also increased the neutrophil count in the peritoneal cavity, demonstrating that SNAPPs can also enhance host cell innate immunity (though the role of the recruited neutrophils was unclear).

Further experimentation revealed that the degree of polymerisation (DP, arm length) and the number of arms on the SNAPPs can modulate their minimum membrane disruptive concentration (MDC), MIC, and MBC values (Shirbin et al., 2018). When tested against *E. coli*, SNAPPs with 4 arms but varying DP showed that increasing DP resulted in lower MIC values. Similarly, SNAPPs with medium arm length (DP of ∼14 repeat units) but a different number of arms showed that an increase in arm number also decreased the MIC value (Table 2). Increasing activity (due to increases in DP and arm number) did correlate with an increase in cytotoxicity, but when tested *in vivo* in a murine model, no systemic organ damage was observed. The IC_50_ values *in vitro* were consistently higher than the MICs with S16_M_ showing the highest TI of 8.6 and S4_L_ a close second with a TI of 8.0 (based on its MIC for *E. coli*), indicating that a balance between activity and biocompatibility is important when determining the therapeutic use of new compounds.

Another star-shaped molecule using hyperbranched polyethylenimine (PEI) as the core and poly (l-lysine) (PLL) as the attached antimicrobial component (PEI-*g*-PLL) also exhibited better activity than its linear counterpart and did not include any hydrophobic components (Lu et al., 2019). This study tested 8 and 15-arm star shaped molecules in combination with arms that had DP of 5, 10, 15 or 20. A linear molecule (linear PLL) with a molecular weight close to that of an 8 arm and 10 DP star-shaped PLL (P2) was also tested. The P2 molecule showed consistently better activity against methicillin-resistant *S. aureus* (MRSA), methicillin-resistant *S. epidermidis* (MRSE), *P. aeruginosa* and *A. baumannii* compared to its linear PLL counterpart. All star-shaped PLLs showed better activity towards Gram-positive than Gram-negative bacteria, as previously noted with the S-GSA molecules that also lacked any hydrophobic groups (Table 2). Arm number and length also impacted the antimicrobial activity of these star PLLs. Converse to SNAPPs, the 8 arm molecules generally had lower MIC values than 15 arm molecules with the same DP. However, like SNAPPs, an increase in DP also resulted in a lower MIC. As expected, all of these star molecules and the linear PLL showed negligible haemolysis due to the lack of hydrophobic groups. P2 was also more resistant to proteolytic degradation by trypsin compared to linear PLL. After a 3-h incubation only 13.5% degradation was noted for P2 compared to 67.3% for linear PLL, demonstrating again how this star-architecture improves stability. The lack of hydrophobic components contributes to improved biocompatibility of these compounds but star-molecules lacking these groups also tend to show preference for Gram-positive over Gram-negative bacteria.

According to the Centers for disease Control and Prevention, Gram-negative bacteria make up three out of the five pathogens that are considered urgent threats and seven out of the eleven that are considered serious threats (Frieden, 2019). As such, targeting Gram-negative bacteria, by including hydrophobic groups and finding a balance between activity and biocompatibility, is an important step in developing broad spectrum antimicrobials into therapeutic compounds.

#### Self-Assembling Peptides as Multimers

Self-assembling of antimicrobials is another approach shown to create multimerised compounds (Figure 3E). This topic has been extensively and recently reviewed by Tian et al. (2015), Malekkhaiat Hä ffner and Malmsten, 2018, Lombardi et al. (2019), Zou et al. (2020) and Yang et al. (2021). Briefly, self-assembly occurs through noncovalent interactions and is often influenced by e.g., peptide length, distribution of hydrophobic and charged residues, and the presence of metal ions. This approach modifies the native AMP to alter its chemical characteristics (see Sections 2 and 3, this review) so that under specific environmental conditions the peptides self-assemble/aggregate. These self-assembled multimers can form micelles (DP7-C), nanofibers ((QL)_6_-Melittin + (QL)_6_-K), lamellae (V_4_D) or fibrils (K_3_(FA)_4_K_3_) (Chen Y.-F. et al., 2019; Kornmueller et al., 2018; Sha et al., 2020; Zhang et al., 2018). To aid peptide self-assembly, AMP sequences can be modified or conjugated to self-assembling sequences or hydrophobic moieties (e.g., lipids or alkyl chains). Self-assembly is also affected by the environment such that changes in the pH, temperature or ionic strength can control self-assembly (Wang J. et al., 2016). These self-assembled antimicrobials often display not only increased antibacterial activity, but reduced toxicity and improved stability. For example, the self-assembling peptide (QL)_6_-Melittin + (QL)_6_-K exhibited similar antibacterial activity towards *E. coli* but had significantly reduced toxicity to NIH 3T3 cells, compared to melittin (Chen Y.-F. et al., 2019). Another self-assembling peptide A_9_K also exhibited antimicrobial activity towards *E. coli* and *B. subtilis* and could kill cancerous HeLa cells, but displayed minimal toxicity towards NIH 3T3 and human red blood cells (Chen et al., 2012). Some advantages of these self-assembling peptides include improved cell selectivity, sustained AMP release and improved stability.

### Penetrating Biofilms

In addition to targeting a broad spectrum of bacteria with AMPs, it also essential to evaluate their potency against biofilms. When bacteria grow in biofilms, they become harder to kill and can result in chronic infections (Bjarnsholt et al., 2013). Many AMPs and AMP-like compounds are only tested against planktonic bacteria *in vitro* under conditions that do not mimic the *in vivo* host environment. Already it has been shown that physiological salts and proteins can negatively affect AMP activity and not all studies explore whether AMPs are effective against biofilms or under physiologically relevant conditions, resulting in poor translation from *in vitro* models to *in vivo* ones. Those that have explored biofilm inhibition indicate that some AMPs do possess antibiofilm activity and that multimerisation can improve this inhibitory action.

Single-Chain Polymeric Nanoparticles (SCPN) that fold into a circular structure with extending oligoethylene glycol (OEG) arms are a type of branched multivalent molecule that are effective in killing *P. aeruginosa* planktonic cells and biofilm (Nguyen et al., 2017). These SCPN compounds are made of amine and hydrophobic groups polymerised at a molar ratio of 2.5:1 along with OEG to create a linear random copolymer that collapses into a micelle when in water. Two of these SCPNs, P_Dab-EH_ and P_Dab-F_, were identified as lead compounds with MIC values of 1.4–2.8 µM and 2.8–5.6 µM against *P. aeruginosa,* respectively, and showed good biocompatibility with H4IIE cells and sheep red blood cells. These compounds were also tested against *P. aeruginosa* PAO1 biofilms at 4 x MIC and CFUs within the biofilm were determined by a drop-plate method. This revealed a 2.6 and 3.3 log_10_ reduction in biofilm CFUs, respectively, compared to an untreated control after 60 min incubation. Colistin was also tested at 4 x MIC for 60 min but only resulted in <1 log_10_ reduction. These compounds were also shown to disperse approximately 72% of pre-formed biofilm mass after a 60 min incubation period as determined by a reduction in biofilm biomass via crystal violet staining.

Another AMP dendrimer consisting of Arg-Trp-Arg and Arg-Tbt-Arg (2) (Tbt: β-(2,5,7-tri-*tert*-butylindol-3-yl)alanine) tripeptide branches (called 2D-24), also exhibited antibiofilm activity against *P. aeruginosa* strains PAO1 and PDO300 (Bahar et al., 2015). At a concentration of 20 μM, 2D-24 killed ∼87.8 and ∼81.7% of PAO1 and PDO300 biofilm cells respectively as determined via a drop-plate method and live/dead staining using membrane permeable dye SYTO9 and membrane impermeable dye propidium iodide. However, no linear compounds were used as a control. The previously mentioned (RW)_4D_ dendrimer also showed antibiofilm activity towards *E. coli* RP473 (Hou et al., 2009). Biofilm formation on a polystyrene surface was determined by crystal violet staining and was reduced by ∼21.8, 47.1, 84.4 and 93.5% by 5, 10, 20 and 40 µM of (RW)_4D_ respectively after 24 h incubation compared to a 0 µM control. The aforementioned multivalent AMPs do not have linear analogues to be compared to and with many linear AMPs also showing antibiofilm activity, it is difficult to determine to what extent multimerisation affects antibiofilm activity.

The previously mentioned lin-SB056-1 AMP, however, is an example where the effect of multimerisation on biofilm formation has been demonstrated. The dendrimeric form of this peptide was again synthesised, but this time without the hydrophobic tail, to create (lin-SB056-1)_2_-K and was tested against *P. aeruginosa* PAO1 biofilm in *in vivo*-like lung epithelial cell and artificial wound models (Grassi et al., 2019). A combination of lin-SB056-1 and EDTA was also tested as a comparison and combinatorial treatment. EDTA has been shown to break up the biofilm extracellular matrix and disrupt the outer membrane of Gram-negative bacteria, making them more susceptible to AMPs (Banin et al., 2006). At concentrations that caused less than 20% haemolysis and epithelial cell death (lin-SB056-1)_2_-K (at 19.25 µM) proved more effective at reducing the number of biofilm-forming *P. aeruginosa* (determined via drop-plate method and subsequent CFU count) than 38.5 µM of its linear counterpart combined with 0.3 mM EDTA. After 4 h incubation, lin-SB056-1/EDTA resulted in a 1-Log unit reduction of biofilm-forming bacteria whereas (lin-SB056-1)_2_-K resulted in a 2-Log unit reduction.

Another example is the modification of naturally occurring anoplin via substitution of residues 4 and 7 with their D-enantiomers that resulted in a more potent and selective compound named anoplin-D4, 7 (Zhong et al., 2019). This compound was further modified with the addition of different length fatty acids at the N-terminus (C_4, 6, 8, 10 or 12_-D4,7). Additionally, anoplin-D4, 7 was also dimerised using copper click chemistry, the N-terminal residues acetylated, and fatty acid chains added to create J-AA-(C_4, 6, 8, 10 or 12_-D4,7 + D4,7). Increasing fatty acid chain length led to increasing antimicrobial activity with the dimers being even more potent against a variety of Gram-positive and Gram-negative bacteria. These analogues (both linear and dimeric compounds) were tested for antibiofilm formation against *S. aureus* ATCC 25923, *E. coli* ATCC 25922 and *P. aeruginosa* ATCC 27853. All compounds could inhibit biofilm formation in a dose dependent manner as indicated by crystal violet staining and absorbance compared to a control sample (no peptide added). However, they were tested and compared to each other at 0.25, 0.5, 1 and 2 times their MIC values rather than at equal concentrations which makes it difficult to determine to what extent d-amino acids and dimerisation affect biofilm inhibition. Estimates for C_8_-D4,7 and J-AA-(C_8_-D4,7 + D4,7) indicate that at equal concentrations the dimerised compounds are more potent at inhibiting biofilm formation in *S. aureus* and *E. coli* but are less potent towards *P. aeruginosa* biofilm compared to their monomer counterpart.

### Synergistic Effects of Antimicrobial Peptides

#### Synergy Between Antimicrobial Peptides and Antibiotics

Clinical use of AMPs alone as a therapeutic still faces some hurdles. However, AMPs as adjuvants for currently used antibiotics may be the first steppingstone to clinical approval. An antimicrobial polymer made up of oligoethylene glycol, hydrophobic and amine groups was tested alongside ten antibiotics (doxycycline, clarithromycin, azithromycin, gentamicin, tobramycin, ampicillin, amoxicillin, ceftriaxone, colistin and ciprofloxacin) that exhibit different modes of action. Out of this group of ten, the polymer only showed synergy with doxycycline and colistin methanesulfonate when tested against *P. aeruginosa* and *E. coli* (Namivandi-Zangeneh et al., 2019). Individually administered, the polymer and doxycycline had MIC values of 32 μg/ml and 8 μg/ml respectively but when used in combination these values were lowered to 8 μg/ml and 1 μg/ml when tested against *P. aeruginosa* PAO1. Against *E. coli* K12, the MIC lowered from 32 μg/ml to 8 μg/ml for the polymer and from 2 μg/ml to 0.5 μg/ml for doxycycline. The mechanisms involved in this synergy are unclear, but it is believed that the polymer increased the uptake of doxycycline via membrane disruption and acted additively with colistin as both compounds act via membrane disruption. However, there was no explanation as to why synergy was not observed with other antibiotics that also act on internal targets, and this may be a result of the size of the pore formed versus the size of the antibiotic.

### Delivery of Synergistic Molecules

Given that AMPs can act in synergy with each other and antibiotics, it follows that they must somehow be co-delivered. Nanoparticles and self-assembling compounds are currently being explored as not only delivery vehicles, but as compounds that also have inherent antibacterial properties. One such example are the previously mentioned star-shaped PLL molecules (also known as multi-armed poly (ʟ-lysine) (MPLL) molecules) which can be crosslinked with poly (ethylene glycol) (PEG) (MPLL-*alt*-PEG) (Lu et al., 2020). The MPLL polymer is positively charged, allowing it to disrupt bacterial membranes, and when crosslinked with PEG can encapsulate negatively charged drugs and self-assemble into micelles. The authors suggested that the cross-linking improves stability of the micelles, allows for more efficient drug loading, and protects the encapsulated drug from degradation and premature release. They also proposed that the cross-linking improves the antibacterial activity of MPLL as it increases the molecular weight, makes it more stable against proteolysis and increases its *in vivo* half-life. Drug release is then based on the pH of the environment. At physiological pH, the MPLL micelles are positively charged and through electrostatic interactions encapsulated the protein tested (interferon α-2b), which is negatively charged. At infectious sites which have an acidic environment, the charge of the encapsulated protein changes from net negative to net positive whilst the side chains of the MPLL molecules remain positively charged. This change in charging state of the protein weakens its electrostatic interaction with the MPLL-*alt*-PEG molecule and results in accelerated drug release. The release rate of interferon α-2b was measured at varying pH and was shown to be fastest at a lower pH. The antimicrobial activity of MPLL was also tested against MRSA and *P. aeruginosa* and compared to the activity of MPLL-*alt*-PEG. The crosslinked MPLL polymers exhibited better antimicrobial activity compared to MPLL alone, with MIC values of 3.8 and 7.5 µM against MRSA and *P. aeruginosa* respectively compared to 7.5 and 15 µM for MPLL alone. Both compounds also exhibited minimal haemolysis with their HC_50_ values being more than 1,000 μg/ml. Field-emission scanning electron microscopy (FE-SEM) and transmission electron microscopy (TEM) of treated and untreated MRSA and *P. aeruginosa* also revealed that the MPLL-*alt*-PEG molecules inhibited bacteria via a membrane-disruption mechanism.

The previous example demonstrates a possible avenue for co-delivery of an AMP-like compound and an anionic drug based on their electrostatic interaction. However, for synergistic AMPs that are both cationic, an alternative method is required. Nanoparticles can be made from various materials and in addition to inherent antibacterial activity, can also act as a delivery system. Porous silicon nanoparticles (pSiNPs) loaded with the cationic bacterial toxin [KLAKLAK]2 made of d-amino acids (dKK), and the AMP lactoferrin (KCFQWQRNMRKVRGPPVSCIKR), is one example (Kwon et al., 2017). In this study, lactoferrin was grafted to dKK which resulted in LACT-dKK that exhibited stronger antibacterial activity than dKK or LACT alone. This tandem peptide was then loaded in pSiNPs and was shown capable of killing *P. aeruginosa*, exhibited low toxicity towards NIH-3T3 cells and low haemolysis. Mice were also treated with unloaded pSiNPs, peptide-pSiNPs, free peptide or PBS (delivered to their lungs via a catheter) and adverse effects to their respiration observed. Compared to the PBS control, mice injected with the free peptide displayed laboured breathing whereas those injected with the peptide-pSiNPs showed no adverse respiration. However, hemotoxylin and eosin staining of lung sections revealed mild bronchial epithelial damage in mice treated with peptide-pSiNPs. The free peptide further caused substantial damage to the lungs, including sloughing of the bronchial epithelium, bronchitis, and interstitial pneumonitis. The more severe adverse response caused by the free peptide highlights the multifaceted need for drug carriers to act not only as delivery vehicles, but to also improve the safety profile of potential antibacterial. Whilst delivery systems for synergistic compounds are needed for efficient co-delivery, they can also be beneficial for delivery of single compounds. With many AMPs having short *in vivo* half-lives, being susceptible to proteolysis and toxic to mammalian cells, efficient delivery systems can help mitigate these limitations. They can also help antimicrobials penetrate biofilms and reach therapeutic concentrations at the target site. Reviews by Liu et al., Teixeira et al. and Drayton et al. give a more comprehensive overview of current nanotechnologies being explored as drug delivery vehicles (Drayton et al., 2020; Liu et al., 2019; Teixeira et al., 2020).

## Conclusion

Much is known about the general properties of AMPs and how they can be manipulated to render them more potent, but this often comes at a cost of increased toxicity. Additionally, there remain the issues of proteolytic instability and reduced activity under physiological conditions and towards biofilms that need to be overcome. Although similar in many respects, the many differences between AMPs means that the modifications that improve activity in some cannot be applied universally and may have limited or detrimental effects on activity of other AMPs. The examples above highlight how dimerisation is not a solution for all AMPs, and that maximising on each individual feature of natural AMPs – helicity, hydrophobicity, amphipathicity and charge – will not necessarily result in a superior compound. Furthermore, as well as the AMP sequence, the order of the amino acid sequence impacts activity. Sequence modifications that do improve linear peptides, do not always lead to increased improvement when these peptides are multimerised. Multimerisation of weaker, non-modified linear AMPs sometimes results in molecules that are more potent than the multimerised forms of their improved linear analogues. As such, a balance between the different adjustments that can be made to improve AMP activity needs to be achieved.

Research is focusing on structure-activity relationships by altering the overall sequence and structure of AMPs and has shown that multimerisation can result in AMP-like compounds that address the limitations faced by many AMPs. However, many multi-unit AMPs are derived through polymer chemistry, as polymerisation is generally faster and cheaper than peptide synthesis. However, an issue with polymers is that their sequence cannot be controlled, the final structure is not precise and there is only partial control over polymerisation (Kuroda and Caputo, 2013). This leads to a range in their molecular weight; a major issue in drug development and regulation since defined molecular weight materials are required for clinical use. Thus, there is a need for future research to address the ideas behind these architecturally unique compounds and create chemically defined molecules that have the potential to overcome the shortcomings of natural AMPs.
