# Supplementary material for: The Potential of Modified and Multimeric Antimicrobial Peptide Materials as Superbug Killers
Source: Front Chem. 2022 Jan 10;9:795433. doi: 10.3389/fchem.2021.795433 (PMC8785218; doi:10.3389/fchem.2021.795433)
Supplement: Supplementary file 1 [file DataSheet1.pdf]

Appendix.

The Potential of Modified and Multimeric Antimicrobial Peptide Materials as Superbug Killers

Tamara Matthyssen [1], Wenyi Li [1], James A. Holden [2], Jason C. Lenzo [2], Sara Hadjigol [1], and Neil M. O'Brien-Simpson [1,*]

[1]ACTV Research Group, The University of Melbourne, Melbourne Dental School, Centre for Oral Health Research, Royal Dental Hospital, Melbourne, Victoria, Australia.

[2]Centre for Oral Health Research, The University of Melbourne, Melbourne Dental School, Royal Dental Hospital, Melbourne, Victoria, Australia

*** Correspondence:**
Neil O'Brien-Simpson
neil.obs@unimelb.edu.au

Appendix A. Antimicrobials and their peptide sequence/polymer composition

Antibacterial	Sequence	Microorganisms tested against											+ net charge	ref.
		Gram-negative bacteria					Gram-positive bacteria							
		E. coli	*K. pneumoniae*	*A. baumannii*	*P. aeruginosa*		*S. aureus*	MRSA	*E. faecalis*	*S. epidermidis*	*B. subtilis*	*E. faecium*		
**PEPTIDES**														
**Tachyplesin-1** [a, e, f]	KWCFRVCYRGICYRRCR	+	+	+	+		+				+	+	6	(Edwards et al., 2016)
**Protegrin-1** [a, e, f]	RGGRLCYCRRRFCVCVGR	+	+	+	+		+				+	+	6	
**Arenicin-3** [a, e, f]	GFCWYVCVYRNGVRVCYRRCN	+	+	+	+		+				+	+	4	
**Polyphemusin-1** [a, e, f]	RRWCFRVCYRGFCYRKCR	+	+	+	+		+				+	+	7	
**Gomesin** [a, e, f]	ZCRRLCYKQRCVTYCRGR	+	+	+	+		+				+	+	6	
**Thanatin** [a, e, f]	GSKKPVPIIYCNRRTGKCQRM	+	+	+	+		+				+	+	6	
**PGLa** [a, f]	GMASKAGAIAGKIAKVALKAL-NH_2	+			+		+						5	(Blazyk et al., 2001; Jin et al., 2005)
**(KIAGKIA)$_3$-NH_2** [a, f]	KIAGKIAKWAGKIAKIAGKIA-NH_2	+			+		+						7	
**(KIGAKI)$_3$-NH_2** [a, f]	KIGAKIKWGAKIKIGAKI-NH_2	+			+		+						7	
**N6** [a]	GFAWNVCVYRNGVRVCHRRAN	+			+		+						4	(Yang et al., 2017)
**peptide P** [a, f]	KWKSFLKTFKSLKKTVLHTLLKAISS	+			+								7	(Huang et al., 2014)
**Magainin 2** [b, c, f]	GIGKFLHSAKKFGKAFVGEIMNS	+	+				+		+			+	4	(Lorenzón et al., 2016)
**helical peptoid mimic of magainin 2**[a, c, f]	(*N*Lys-*N*spe-*N*spe)$_4$	+					+						4	(Nam et al., 2020)
**V$_{681}$** [a]	KWKSFLKTFKSAVKTVLHTALKAISS												6	(Zhang et al., 1998)
**V16L** [a, f]	KWKSFLKTFKSAKKTLLHTALKAISS	+			+								7	(Tan et al., 2014)
**V13K$_L$** [a, f]	KWKSFLKTFKSAKKTVLHTALKAISS	+			+		+			+		+	7	(Jiang et al., 2008)
**C18G** [a, d]	ALWKKLLKKLLKSAKKLG	+			+		+			+			7	(Saint Jean et al., 2018)
**AR-23** [a, d, f]	AIGSILGALAKGLPTLISWIKNR	+	+		+		+			+	+	+	4	(Zhang et al., 2016)
**lin-SB056-1** [b, f]	KWKIRVRLSA	+			+		+		+				5	(Manzo et al., 2015)
**6K-F17** [a, f]	KKKKKKAAFAAWAAFAA-NH_2	+			+								7	(Stone et al., 2019)
**1KAMP** [a, f]	KAAKKFAKAWAKAFKAA-NH_2	+			+								7	
**6K-F17-4L** [a, f]	KKKKKKALFALWLAFLA-NH_2	+			+								7	

**1KAMP-4L** [a, f]	KALKKFLKAWAKLFKAL-NH_2	+			+								7	
**Aurein 1.2** [a, f]	GLFDIIKKIAESF	+			+		+		+				1	(Migoń et al., 2019)
**Aurein M2** [a, c, d, f]	GLFKIIKKIAKSF-NH_2	+			+		+		+				5	(Ramezanzadeh et al., 2021)
**Aurein M3** [a, c, d, f]	GLFKIIKKIWKSF-NH_2	+			+		+		+				5	
**aurein 2.2-Δ3** [a, f]	GLFDIVKKVVGAL-NH_2						+						2	(Kumar et al., 2019; Raheem et al., 2020)
**peptide 77 (Arg and Trp rich aurein 2.2-Δ3)** [a, f]	RLWDIVRRWVGWL-NH_2						+						3	
**BP100** [a, c, d, f]	KKLFKKILKYL	+	+		+		+					+	6	(Torcato et al., 2013)
**R-BP100** [a, c, d, f]	RRLFRRILRYL	+	+		+		+					+		
**α-MSH (6-13)** [a, c, f]	HFRWGKPV						+	+					2	(Singh et al., 2020)
**Ana-5** [a, c, f]	RWRWWKPV						+	+					3	
**Temporin-1CEb** [a, f]	ILPILSLIGGLL-NH_2	+	+		+		+		+			+	1	(Shang et al., 2012)
***L*-K6** [a, f]	IKKILSKIKKLLK-NH_2	+	+		+		+		+			+	7	
**A3-APO** [a, e]	Chex-RPDKPRPYLPRPRPPRPVR	+	+				+						6	(Li et al., 2016; Otvos et al., 2005, 2014)
**ᴅL5** [a, c]	KLKLLLLLKLK-NH_2	+					+						4	(Manabe & Kawasaki, 2017)
**GN-4 peptide**	RWKKWWRWL-NH_2	+			+		+						5	(Mojsoska et al., 2015)
**GN-4 peptoid**	*N*lys-*N*trp-*N*lys-*N*lys-*N*trp-*N*trp-*N*lys-*N*trp-*N*leu-NH_2													
**W^6-Hy-al** [a, f]	IFGAIWPLALGALKNLIK-NH_2	+			+		+				+		3	(Crusca et al., 2011)
**LF11** [a]	FQWQRNIRKVR-NH_2	+											5	(Zweytick et al., 2011)
**LF11-215** [a]	FWRIRIRR-NH_2	+											5	
**KR12** [a, d, f]	KRIVQRIKDFLR-NH_2		+	+	+		+					+	6	(Kamysz et al., 2020)
**Anoplin-D4, 7** [a, d, f]	GLLkRIkTLL-NH_2	+	+		+		+	+			+		4	(Zhong et al., 2019)
**MSI-103** [a, f]	$[KIAGKIA]_3$-NH_2	+			+		+		+				7	(Gagnon et al., 2017)
**Distinctin** [a]	Chain A - ENREVPPGFTALIKTLRKCKII	+			+		+		+				7	(Batista et al., 2001)
	Chain B - NLVSGLIEARKYLEQLHRKLKNCKV													
**PST13-RK** [a, d]	KKKFPWWWPFKKK-NH_2	+			+		+				+		7	(Yang et al., 2009)
**RLYR dendrimer arm** [a (zone of inhibition), f]	RLYR	+			+		+		+				2	(Tam et al., 2002)
**$(RW)_n$-NH_2 series** [a, c, f]	$(RW)_n$	+					+						1 + n	(Z. Liu, Brady, et al., 2007; Z.

														Liu, Young, et al., 2007)
**2D-24** [c, f]	RWR and R-β (2,5,7-tri-tert-butylindol-3-yl)alanine-R				+								positive	(Bahar et al., 2015)
**DP7-C**	VQWRIRVAVIRK-cholesterol				+			+					4	(R. Zhang et al., 2018)
**$(QL)_6$-Mel + $(QL)_6$-K** [a, d]	HHHQLQLQLQLQLQL-GIGAVLKVLTTGLPALISWIKRKRQQ + WKKQLQLQLQLQLQLKK	+											positive	(W. Chen et al., 2019)
**V_4D**	VVVVD													(Kornmueller et al., 2018)
**$K_3(FA)_4K_3$** [a, d, f]	KKKFAFAFAFAKKK	+											6	(Sha et al., 2020)
**A_9K** [c, d, f]	AAAAAAAAAK	+									+		1	(C. Chen et al., 2012)
**POLYMERS**														
**Poly-β-peptides** [a, c, d, f]	3-tert-Butyloxycarbonylaminomethyl-4,4-dimethyl azetidin-2-one (DM) and 4-Butyl-azetidine-2-one (Bu)	+	+	+	+		+			+				(Q. Zhang et al., 2019)
**DLL, BLG polymer** [a, d, f]	*Nε-tert*-butyloxycarbonyl-ᴅ,ʟ-lysine and γ-benzyl-L-glutamate				+									(Jiang et al., 2020)
**PEA copolymers** [c, d, f]	di-*p*-nitrophenyl adipate and p-toluenesulfonic acid salt of bis-L-lysine/arginine/phenylalanine	+					+						positive	(J. Zhu et al., 2019)
**bis-MPA umbrellas** [a, c, e, f]	2,2 bis(hydroxymethyl) propionic acid and Boc-*β*-alanine	+					+							(A. Chen et al., 2020)
**Glucosamine-Functionalised Star Polymers** [a, d, f]	polyglucosamine and polylysine	+			+		+	+	+					(Wong et al., 2016)
**SNAPPs** [a, c, f]	ʟ-lysine(Z)–NCA and ᴅʟ-valine–NCA onto poly(amido amine) core	+	+	+	+		+							(Lam, O'Brien-Simpson, et al., 2016)
**PEI-*g*-PLL** [a, c, d, f]	polyethylenemine core and poly(ʟ-lysine) arms			+	+			+					positive	(Lu et al., 2019)
**SCPN-OEG, P_{Dab-EH} and P_{Dab-F}** [a, d, f]	oligoethylene glycol, *tert*-butyl (2-acrylamidoethyl) carbamate or 2-Phenylethyl acrylate	+			+									(Nguyen et al., 2017)

a – MIC determined by broth microdilution and measuring optical density
b – MIC determined by broth microdilution and visual analysis after addition of resazurin
c – MBC determined by broth microdilution and subculturing onto agar plates
d – Cytotoxicity determined by formazan production and absorbance measurement
e – Cytotoxicity determined via Alamar or trypan blue assay
f – Haemolytic activity determined by measuring haemoglobin release from red blood cells
